# Genome-scale analysis of the genes that contribute to *Burkholderia pseudomallei* biofilm formation identifies a crucial exopolysaccharide biosynthesis gene cluster

**DOI:** 10.1371/journal.pntd.0005689

**Published:** 2017-06-28

**Authors:** Grace I. Borlee, Brooke A. Plumley, Kevin H. Martin, Nawarat Somprasong, Mihnea R. Mangalea, M. Nurul Islam, Mary N. Burtnick, Paul J. Brett, Ivo Steinmetz, David P. AuCoin, John T. Belisle, Dean C. Crick, Herbert P. Schweizer, Bradley R. Borlee

**Affiliations:** 1 Department of Microbiology, Immunology and Pathology, Colorado State University, Fort Collins, Colorado, United States of America; 2 Department of Molecular Genetics and Microbiology, University of Florida, Gainesville, Florida, United States of America; 3 Department of Microbiology and Immunology, University of South Alabama, Mobile, Alabama, United States of America; 4 Institute of Hygiene, Microbiology, and Environmental Medicine, Medical University of Graz, Graz, Austria; 5 Department of Molecular Microbiology and Immunology, University of Nevada-Reno, School of Medicine Reno, Nevada, United States of America; Mahidol University, THAILAND

## Abstract

*Burkholderia pseudomallei*, the causative agent of melioidosis, is an important public health threat due to limited therapeutic options for treatment. Efforts to improve therapeutics for *B*. *pseudomallei* infections are dependent on the need to understand the role of *B*. *pseudomallei* biofilm formation and its contribution to antibiotic tolerance and persistence as these are bacterial traits that prevent effective therapy. In order to reveal the genes that regulate and/or contribute to *B*. *pseudomallei* 1026b biofilm formation, we screened a sequence defined two-allele transposon library and identified 118 transposon insertion mutants that were deficient in biofilm formation. These mutants include transposon insertions in genes predicted to encode flagella, fimbriae, transcriptional regulators, polysaccharides, and hypothetical proteins. Polysaccharides are key constituents of biofilms and *B*. *pseudomallei* has the capacity to produce a diversity of polysaccharides, thus there is a critical need to link these biosynthetic genes with the polysaccharides they produce to better understand their biological role during infection. An allelic exchange deletion mutant of the entire *B*. *pseudomallei* biofilm-associated exopolysaccharide biosynthetic cluster was decreased in biofilm formation and produced a smooth colony morphology suggestive of the loss of exopolysaccharide production. Conversely, deletion of the previously defined capsule I polysaccharide biosynthesis gene cluster increased biofilm formation. Bioinformatics analyses combined with immunoblot analysis and glycosyl composition studies of the partially purified exopolysaccharide indicate that the biofilm-associated exopolysaccharide is neither cepacian nor the previously described acidic exopolysaccharide. The biofilm-associated exopolysaccharide described here is also specific to the *B*. *pseudomallei* complex of bacteria. Since this novel exopolysaccharide biosynthesis cluster is retained in *B*. *mallei*, it is predicted to have a role in colonization and infection of the host. These findings will facilitate further advances in understanding the pathogenesis of *B*. *pseudomallei* and improve diagnostics and therapeutic treatment strategies.

## Author summary

*B*. *pseudomallei*, the etiological agent of melioidosis, is an emerging pathogen with limited therapeutic options and no available vaccines. A better understanding of the role of biofilm formation during pathogenesis will aid in melioidosis diagnosis and the development of new therapeutics and vaccines. Melioidosis has both acute and chronic disease manifestations in addition to a highly variable period of latency, which contributes to complications in diagnosis and treatment of the disease. Relapsing melioidosis is correlated with biofilm formation and the role of biofilm growth during chronic human infections has been widely accepted. We utilized a two-allele sequence defined transposon mutant library of *B*. *pseudomallei* 1026b to identify genes involved in biofilm formation. This study identified factors that contribute to biofilm formation and included a previously undescribed exopolysaccharide and the genes underlying its biosynthesis. Since antibiotic tolerance in *B*. *pseudomallei* has been associated with biofilm formation, the genes identified in this study that contribute to biofilm production are potential targets for therapeutic development. Additionally, the products of these biofilm genes could be used for the development of diagnostics and vaccines.

## Introduction

*B*. *pseudomallei*, an environmental saprophyte, is the etiological agent of melioidosis and has been traditionally described as being endemic to Northern Australia and Southeast Asia [1]. However, an increasing body of evidence indicates *B*. *pseudomallei* is more widely distributed than previously thought [2–4]. As diagnostics and clinical awareness improve, melioidosis cases and their bacterial cause are increasingly detected worldwide [5]. *B*. *pseudomallei* is an important global pathogen, as indicated by a recently published study that predicts approximately 165,000 human cases of melioidosis with greater than 50% mortality annually in 79 countries where the pathogen is probably endemic [6]. Due to the lack of vaccines, the intrinsic resistance to numerous antibiotics, and high mortality rate associated with acute infections, in addition to its potential use as an agent for biological warfare and bioterrorism, *B*. *pseudomallei* is currently designated as a Tier 1 select agent by regulatory agencies in the United States [7, 8].

*B*. *pseudomallei* is well known for its ability to produce biofilm, which may be critical to the increased persistence of this pathogen in the environment [9]. Bacteria growing as a biofilm are embedded in a matrix comprised of self-produced extracellular polymeric substances (EPS) that include polysaccharides, proteins, lipids, and nucleic acids. This matrix is thought to serve as a scaffold to hold biofilm cells together and protect from some antimicrobials (see [10] for recent review). Despite the importance of the EPS components that comprise the biofilm matrix, we know surprisingly little about it [11]. EPS from *B*. *pseudomallei* has been described for capsular polysaccharides, O-polysaccharides, and exopolysaccharides (for review [12]). However, the contribution and characterization of these EPS components to *B*. *pseudomallei* pathogenesis has not been conclusively evaluated in chronic models of melioidosis. Additional capsular polysaccharides and exopolysaccharides also remain to be identified and characterized. In the absence of information linking the identity, structural composition, and expression of these EPS components, it will not be possible to determine their role in the establishment and progression of disease.

Multiple polysaccharides associated with the surface of *B*. *pseudomallei* have been characterized based on structure and antigenicity. Two of the best characterized EPS components are the primary capsule (CPSI, Bp1026b_I0499-Bp1026b_I0524) [13, 14] and >150 kDa acidic exopolysaccharide [15, 16]. Additional biosynthetic clusters have also been identified that are predicted to encode three additional capsules (CPSII-IV) [12]. However, the composition, structure, and role during pathogenesis is not well understood for all of these polysaccharides. Capsule III gene expression has been shown to be increased in water as compared to relatively low levels of expression *in vivo*, which is proposed to contribute to the survival of *B*. *pseudomallei* in the environment [17]. However, the role of this capsule in *B*. *pseudomallei* is unknown. CPSI was originally described as O-antigenic polysaccharide (O-PS I) and is an unbranched homopolymer consisting of monosaccharide repeats having the structure [→3)-2-O-acetyl-6-deoxy-β-D-*manno*-heptopyranose-(1→] [13, 14]. The structure of an acidic exopolysaccharide has also been reported to be a unique linear tetrasaccharide repeating unit consisting of three galactose residues and one 3-deoxy-D-*manno*-2-octulosonic acid (Kdo) residue [15, 16].

A number of published studies have identified genes involved in the production of the *B*. *pseudomallei* biofilm matrix [18–24]. However, the contribution of these genes to pathogenesis in *B*. *pseudomallei* has been complicated by the use of multiple strains and different genetic approaches, which has resulted in conflicting reports of the role of key biofilm matrix components [25–27]. To gain a more comprehensive understanding of the genes that contribute to biofilm formation, we screened a sequence-defined two-allele library of transposon mutants comprising approximately 81% coverage of ORFs in *B*. *pseudomallei* 1026b, which is a clinical isolate from a diabetic patient afflicted with disseminated melioidosis [28]. This strain has become a model strain for *B*. *pseudomallei* studies of pathogenesis and antibiotic resistance because the genome is fully sequenced, publicly available, amenable to genetic analysis, and naturally transformable [28–30]. Numerous animal models have also been developed to study acute and chronic disease associated with melioidosis [10, 31–34].

In this systematic analysis of genes that contribute to biofilm production, we identified 59 transposon insertion mutants in unique genetic loci that have an integral role in *B*. *pseudomallei* 1026b biofilm formation. These loci encode polysaccharide biosynthesis, fimbriae, motility, cellular homeostasis, transport, and hypothetical genes. One of the key EPS components discovered in this study is synthesized by a novel 28 kb biosynthesis gene cluster (Bp1026b_I2907-Bp1026b_I2927), which we have designated as *becA-R* (*b**iofilm* *e**xopolysaccharide gene* *c**luster*). In addition to identifying these genetic loci as requirements for biofilm formation, we evaluated transposon insertion mutants in *B*. *pseudomallei* genes previously described to contribute to biofilm formation. This is the first report that describes the multiple genetic components that contribute to biofilm formation on a genome-wide scale in *B*. *pseudomallei*.

## Methods

### Growth conditions

All experiments were performed in the BSL3 facility at Colorado State University except for studies conducted with the select agent excluded *B*. *pseudomallei* strain Bp82, which was handled at BSL2. Transposon (T24) mutant derivatives described in these studies (S1 Table) were generated during the production of a comprehensive two-allele sequence defined transposon mutant library of *B*. *pseudomallei* 1026b (manuscript in preparation). Briefly, the *B*. *pseudomallei* 1026b two-allele library contains two mutants per gene for which the transposon locations were confirmed by resequencing. Two different representative mutants were chosen from the primary library with transposon insertion sites between 5% and 80% of the respective predicted open reading frame, and for which the precise transposon-genome junctions have been determined by sequencing. T24 is a Tn*5*-derived transposon containing a select agent approved kanamycin resistance selection marker that was constructed in the laboratory of Colin Manoil (University of Washington) (http://www.gs.washington.edu/labs/manoil/transposons/transposons.pdf). Transposon mutants in the primary biofilm screen were grown in 1.2 mL LB with 10% glycerol and 35 μg/mL kanamycin. For all subsequent assays, overnight cultures of selected transposon mutants were grown in LB (10 g/L tryptone, 5 g/L yeast extract, and 5 g/L NaCl) with 300 μg/mL kanamycin. Location of the transposon insertion was reconfirmed by sequencing. Swimming motility and growth curve assays were conducted as previously described [35]. For growth on NAP-A plates [36], overnight cultures were either pin replicated or spotted (3 μL) and incubated at 37°C for two days. All strains and plasmids are described in S2 Table.

### Primary and secondary biofilm screens

The primary biofilm screen was conducted using deep 96 well plates (Simport #T110-10S). Plates were inoculated with a 96-well pin replicator and statically incubated at 37°C for two days. Transposon insertional mutants that visually appeared to have reduced or no pellicle formation were selected and plated on LB kanamycin plates for colony isolation. Biofilm phenotypes were further evaluated in static microtiter biofilm assays as previously described [35]. All transposon insertions were confirmed via Sanger sequencing. The DOOR 2.0 operon database was used to identify the first gene in each operon using *B*. *pseudomallei* K96243 as the reference genome [37].

### Comparative analysis of biofilm-associated exopolysaccharide gene cluster

A combination of bioinformatics tools and open-access genomic databases was used to compare the putative exopolysaccharide gene clusters from the sequenced genomes of *B*. *pseudomallei* 1026b (taxid: 884204), *B*. *cenocepacia* J2315 (taxid: 216591), *B*. *vietnamiensis* G4 (taxid: 269482), *B*. *mallei* ATCC 23344 (taxid: 243160), and *B*. *thailandensis* E264 (taxid: 271848). Regions of homology were initially identified using BLASTN (BLAST, NCBI) using default parameters and the Burkholderia Orthologous Groups classification system from the Burkholderia Genome Database (http://www.burkholderia.com, [38]). Genome sequences for *B*. *pseudomallei* 1026b chromosome I (accession number: NC_017831.1) and *B*. *cenocepacia* J2315 chromosome II (accession number: NC_011001.1) were downloaded from the GenBank sequence database (NCBI, NIH) and regions of interest were extracted using Geneious version 7.1.7 (http://www.geneious.com, [39]). Comparative analysis of *bce-I* and *bce-II* gene clusters was conducted for *B*. *pseudomallei* and *B*. *vietnamiensis* G4. Previously published research on cepacian production in several *Burkholderia* spp. strains has linked polysaccharide production and structural characterization to specific biosynthetic gene clusters that were annotated in the *B*. *vietnamiensis* G4 genome [40]. GenBank sequence files were visualized with EasyFig version 2.2.3 [41] and Python programming language version 2.7 (http://www.python.org). Homology and inversions among gene loci were calculated using BLASTN with the EasyFig default parameters of a minimum identity cutoff of 60%. Individual percent identities for each locus were calculated using Multiple Sequence Comparison by Log-Expectation (MUSCLE) tool provided by the European Bioinformatics Institute (EMBL-EBI), which creates percent identity matrices using Clustal 2.1 [42]. To calculate and visualize sequence homology, we used a threshold E-value of 1e-3 and minimum identity value of 0.60 for blast hits drawn. Cut-off thresholds were validated using the command line BLAST+ application to generate a frequency distribution of E-values for all predicted homologous alignments and false-positive non-homologs.

### Deletion of biofilm-associated exopolysaccharide gene cluster

Construction of EPS biosynthetic cluster deletions was accomplished by amplification and fusion of flanking genomic sequences external to the region of interest using SOEing PCR and cloning into the allelic exchange vector, pEXKm5 [43]. Introduction of the suicide vector for allelic exchange was accomplished by conjugation of *E*. *coli* RHO3 with pEXKm5 constructs into *B*. *pseudomallei* [43]. The mutations were verified using internal and external PCR primers to the gene of interest, after counterselection and screening for *B*. *pseudomallei* kanamycin-sensitive clones containing the putative deletion mutation. PCR primers were designed using genomic sequence obtained from the Burkholderia Genome Database [38]. SOEing PCR used the following primers: left flank (5’- NN**CCCGGG**CGAACAGGTTGCGCGGACGGT-3’) and (5’- ACGAACGACGACAGCCGCCGTCCCGCGCGGACCTCAGAAGC-3’) and right flank with (5’- GCTTCTGAGGTCCGCGCGGGACGGCGGCTGTCGTCGTTCGT-3’) and (5’- NNN**CCCGGG**AAGAGCCTCGCGACCGCGCAC-3’) to amplify the regions flanking Bp1026b_I2907-Bp1026b_I2927. Primers incorporated XmaI sites as indicated in bold text. The 1.5 kb flanking region was cloned into pEXKm5 and introduced into *E*. *coli* RHO3 for allelic exchange in *B*. *pseudomallei* 1026b and *B*. *pseudomallei* 1026b Δ*wcbR-A*::*FRT-Zeo* and into the attenuated *B*. *pseudomallei* Bp82 and *B*. *pseudomallei* Bp82 Δ*wcbR-A*::*FRT-Zeo* [44].

### Complementation of I1954::T24, I2907::T24 (*becA*), and II2527::T24

Complementation studies utilized a transposase-mediated integration approach for conditional expression of full length Bp1026b_I1954, Bp1026b_I2907, and Bp1026b_II2527 under the control of an inducible P_*tac*_ promoter to allow for controlled expression in *B*. *pseudomallei* as previously described [35]. Gene expression constructs were introduced into the chromosome using pUC18T-mini-Tn*7*T-Km-LAC [45]. Full length genes were amplified from genomic *B*. *pseudomallei* Bp82 DNA using the following primers: 5’-NNN**CCCGGG**ATGGATTTCGTTTTGCGGG-3’ and 5’-NN**AAGCTT**TCACTCCGCGTCCCCCTG-3’ for Bp1026b_I1954, 5’-NNN**CCCGGG***AGGAGGATATTC*ATGAATCTGTCTTCCCCGTTATCC-3’ and

5’-NNN**AAGCTT**TCAATCGAGCGCGCGCA-3’ for Bp1026b_I2907 (*becA*), and

5’-NNN**CCCGGG**ATGACGCCCGAACGGCCCGACGCTT-3’ and 5’-NNN**AAGCTT**TCAATCCTCATGCCCCGCGA-3’ for Bp1026b_II2527 with engineered XmaI and HindIII sites (in bold) and a ribosomal binding site in italics for Bp1026b_I2907. Amplified PCR products were cloned into pUC18T-miniTn*7*T-Km-LAC and sequences were verified by Sanger sequencing. The T24 transposon which contains a kanamycin resistance cassette was removed from I1954::T24, I2907::T24 (*becA*), and II2527::T24 using a FLP-mediated recombinase strategy that leaves behind a *FRT* signature sequence in the disrupted gene. Triparental mating, Tn*7* integration, and validation of complementation constructs was done as previously described [35]. Mini-Tn*7* insertion was confirmed to be at the *glms2* neutral site. Conditional expression of Bp1026b_I1954, Bp1026b_I2907, and Bp1026b_II2527 in *B*. *pseudomallei* was achieved by adding 1mM isopropyl-β-D-thio-galactopyranoside (IPTG) to the growth medium.

### Exopolysaccharide isolation and preparation

*B*. *pseudomallei* Bp82 cultures were grown in LB supplemented with 80 μg/mL adenine for 16 h. 400 μL of the overnight cultures was spotted onto a polycarbonate 47 mm 0.2 μm (Poretics) membrane disk placed on NAP-A plates for a total of four membranes per culture and incubated at 37°C for 24 h. The membranes were transferred onto fresh NAP-A plates and incubated at 37°C for another 24 h. Membranes were placed in 50 mL conical tubes containing 20 mL 1X PBS and vortexed for 30 min. Exopolysaccharide extraction was performed as described by Steinmetz et al. with the exception of the initial growth of cells on NAP-A plates [15]. Briefly, cells were centrifuged for 4 h at 20,000g at 4°C, and then the supernatant was heated at 80°C for 30 min and centrifuged again for 4 h at 20,000g at 4°C. Supernatant was precipitated with 80% (vol/vol) ethanol overnight at -20°C. Precipitate was centrifuged for 30 min at 3,000g at 4°C, washed with 80% ethanol, centrifuged, and washed with 96% ethanol. Precipitate was solubilized in PBS and treated with RNaseA and DNaseI for 2.5 h and then centrifuged for 30 min at 20,000g at 4°C. Supernatants were precipitated with 80% ethanol and centrifuged. Precipitate was dissolved in LC-MS grade water. The protein concentrations from harvested cells were quantified with the 660nm protein assay kit (Pierce) to estimate biomass prior to exopolysaccharide isolation.

### Western blot analysis of exopolysaccharides and purified capsule

Western blot analysis was performed on semi-purified extracts of polysaccharides from the select-agent exempt *B*. *pseudomallei* Bp82. Exopolysaccharides were purified as described above from cultures of Bp82, Bp82 Δ*becA-R*, Bp82 Δ*wcbR-A*, and Bp82 Δ*becA-R* Δ*wcbR-A*. Exopolysaccharide samples were diluted to normalize loading amounts equivalent to 2 μg of the original biomass based on total protein in samples prior to extraction. To obtain purified *B*. *pseudomallei* CPSI, culture media was inoculated with *B*. *pseudomallei* RR2683 (O-polysaccharide mutant; select agent-exempt strain) and incubated overnight at 37°C with vigorous shaking [14]. Cell pellets were obtained by centrifugation and extracted using a modified hot aqueous-phenol procedure [13]. Purified CPSI was obtained as previously described [14]. Semi-purified exopolysaccharide extracts and purified CPSI were added to Laemmli buffer containing β-mercaptoethanol. Samples were run on 4–15% Criterion gradient gels (Bio-Rad). Gels were transferred to a 0.2 μm PVDF membrane (Bio-Rad) and blocked in 1X TBST containing 5% (w/v) skim milk. Immunoblots were probed with either 1:2,000 primary *B*. *pseudomallei*-specific CPSl (4C4 IgG1) [46] or 1:2,000 acidic exopolysaccharide (mAb 3015) [15] and detected with goat anti-mouse poly HRP secondary (1:50,000) (Pierce). The immunoblots were visualized using a Clarity Western ECL Blotting Substrate. Images were taken with a Biorad ChemiDoc XRS+.

### Analysis of carbohydrate composition

Carbohydrate analysis of *B*. *pseudomallei* polysaccharide extracts was done following a similar approach as previously described with modification [47]. Aliquots of the partially purified *B*. *pseudomallei* Bp82 exopolysaccharide extracts and a mixture of monosaccharide standards (5 μg each, rhamnose, arabinose, ribose, fucose, mannose, glucose and galactose, 100 μg/mL stock solution in water) were spiked with 5 μg of internal standard, 3-O-methylglucose (100 μg/mL stock solution in water) and dried under nitrogen without applying any heat. The dried samples were hydrolyzed for 2 h at 120°C with 2M TFA and alditol acetate derivatives of the resulting monosaccharides were generated. After cooling to room temperature, the hydrolysates were dried under a gentle stream of nitrogen to remove TFA. The resultant monosaccharides were reduced with sodium borodeuteride (10 mg/mL in 1M ammonium hydroxide-ethanol, 1:1) overnight at room temperature. The reaction was terminated with 3–4 drops of glacial acetic acid. The reduction products were dried under nitrogen in the presence of methanol to remove excess borodeuteride and subsequently per-O-acetylated with acetic anhydride at 100°C for 2 h to convert each monosaccharide to its corresponding alditol acetate. The alditol acetates were extracted with a biphasic partition of chloroform and water. The organic phase was dried under nitrogen without applying heat, reconstituted in chloroform and analyzed by GC-MS.

The GC-MS analyses of the alditol acetates were performed with a CP 3800 gas chromatograph coupled with an MS3200 mass spectrometer (Varian Inc. Palo Alto, CA). Helium was used as carrier gas at a constant flow of 1 mL/min. The alditol acetates were chromatographically separated on a VF- 5ms column (30 mm x 0.25 mm i.d. x 0.25 μm film thickness, Agilent J & W). Chromatographic separation of the alditol acetates was achieved with the temperature gradient: 100°C for 1 min., increased to 150°C at 20°C/min., increased to 200°C at 2.5°C/min, and finally increased to 275°C at 30°C/min. Total chromatographic time was 37 min. The mass spectrometer was operated in the EI mode 70 eV with a source temperature 250°C, transfer line temperature 250°C, scan range 50–450 amu. Data acquisition and analysis were ascertained by Varian MS workstation software. Identification of the alditol acetate derivatives was carried out by comparing retention time and mass spectra with authentic standards. The peak area of individual alditol acetates was calculated from the total ion chromatogram, normalized to peak area of the internal standard and finally normalized to the total protein content of sample.

### Statistical analysis

All statistical analyses were performed using GraphPad Prism (GraphPad Software, Inc.). Mutant strains were normalized to wild type. The biofilm data met the assumption of normally distributed values, and therefore a paired Student’s t-test was utilized to analyze the data. The data was not distributed normally for the analysis of swimming motility, thus the Mann-Whitney test was used to analyze the motility data. Significance in all analyses was defined by a calculated p-value less than or equal to 0.001, which was determined using the Bonferroni correction to account for multiple comparisons. Error bars indicate standard error of the mean.

## Results

### Identification of biofilm-deficient mutants

Key genes involved in *B*. *pseudomallei* biofilm formation were identified by screening a 1026b two-allele transposon library for mutants defective in pellicle biofilm formation. This approach allows for efficient genome-scale phenotypic screening of nearly all the non-essential genes to identify putative gene function. During the primary biofilm screen, 118 unique transposon insertional mutants exhibited reduced or no pellicle formation by visual inspection. For secondary screening purposes, we performed a quantitative static biofilm assay with the 118 transposon insertional mutants identified in the primary screen. Sequence analysis was performed to eliminate duplicate transposon insertions in the same ORF. Additionally, establishing a cut-off of >20% decrease in biofilm formation in the quantitative biofilm assay resulted in the retention of 59 mutants for further analyses (S1 Table).

**Fig 1:**
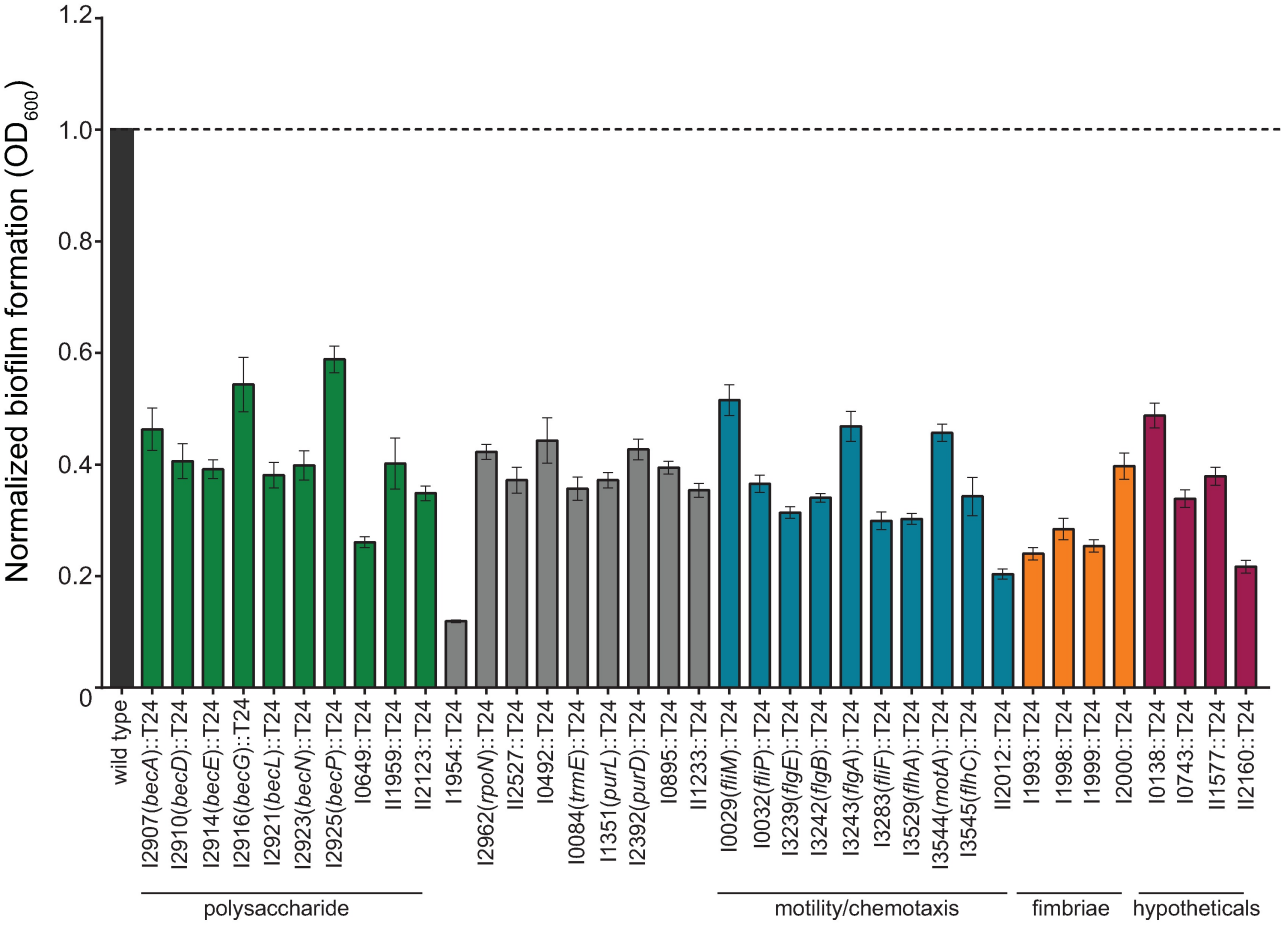
*B*. *pseudomallei* T24 transposon mutants impaired in biofilm formation. The wild type and 37 transposon mutants were grown statically for 24 h at 37°C in polystyrene plates. Biofilm formation was quantified using crystal violet. All transposon mutants exhibited at least a 40% decrease and were tested at least twice in replicates of six. Error bars indicate standard error of the mean.

As a means to reduce the number of transposon insertion mutants in follow-up assays, we also conducted bioinformatics analyses with DOOR 2.0 operon analysis [37]. Based on this analysis, 37 transposon insertional mutants which represented the first gene in each putative operon were selected (S1 Table). These transposon insertion mutants exhibited an approximately 40–60% decrease in biofilm formation as compared to the wild type (Fig 1). A majority of the transposon insertions with a biofilm-deficient phenotype were within genes located in clusters on chromosome I and included genes predicted to produce flagella, fimbriae, regulators, polysaccharides, and an assortment of housekeeping and hypothetical proteins.

Genes involved in the production of flagella and pili are key structural components that are known to contribute to the establishment and initial phases of bacterial biofilm formation in some bacteria (for review [48]). As predicted, the screen identified 17 transposon insertions in genes involved in either motility or chemotaxis that were abrogated in biofilm formation (S1 Table). We chose to characterize ten transposon mutants based on the criteria described above. All of the motility/chemotaxis mutants I0029::T24 (*fliM*), I0032::T24 (*fliP*), I3239::T24 (*flgE*), I3242::T24 (*flgB*), I3243::T24 (*flgA*), I3283::T24 (*fliF*), I3529::T24 (*flhA*), I3544::T24 (*motA*), I3545::T24 (*flhC*), and II2012::T24 were decreased in biofilm formation as compared to the wild type (Fig 1).

The transposon screen also identified pili biosynthesis genes, which are known to play a role in establishing biofilms in some bacteria. The fimbriae gene cluster identified in this screen is one of six predicted type I fimbriae biosynthesis clusters in *B*. *pseudomallei* [49] and is homologous to the *cupE* gene cluster that has been implicated in *P*. *aeruginosa* biofilm formation [50]. All of the genes (Bp1026b_I1992-I2000) in this 11 kb gene cluster were identified in this transposon screen (S1 Table). All four of the representative transposon insertion mutants from this gene cluster were significantly impaired in biofilm formation (Fig 1).

Furthermore, we also identified transposon insertion mutants in four hypothetical genes (Bp1026b_I0138, Bp1026b_I0743, Bp1026b_II1577, and Bp1026b_II2160), one transporter gene (Bp1026b_II1233), two genes involved in purine synthesis (Bp1026b_I1351, *purL*, and Bp1026b_I2392, *purD*), RNA polymerase factor sigma 54 (Bp1026b_I2962, *rpoN*), a DNA response regulator (Bp1026b_I1954), a tRNA modification GTPase (Bp1026b_I0084, *trmE*), a DnaA regulator inactivator Hda (Bp1026b_I0492), a sigma-54 interacting response regulator protein (Bp1026b_I0895), and a sensor histidine kinase/response regulator (Bp1026b_II2527) (S1 Table) that resulted in decreased biofilm formation (Fig 1). The I1954::T24 mutant exhibited a >80% decrease in biofilm formation (Fig 1). Based on bioinformatics analysis with SMART, Bp1026b_I1954 is predicted to possess REC and transcriptional regulator domains and is most likely part of a two-component regulatory system.

One of the more striking findings from this study was the near saturation of a putative 28 kb polysaccharide biosynthesis gene cluster (Bp1026b_I2907-Bp1026b_I2927) which has been designated *becA-R* (*biofilm exopolysaccharide gene cluster*). Only three genes within this novel 18 gene biosynthetic cluster were not identified during the primary phenotypic screen of the transposon library. Of these three, Bp1026b_I2922 (*becM*) and Bp1026b_I2924 (*becO*) are not represented in the two-allele library and were unavailable to test. Bp1026b_I2915 (*becF*) is represented in the library but was not identified in this screen for biofilm deficient mutants. The transposon screen also identified duplicate alleles for eight out of 18 genes in this cluster. This biosynthetic cluster contains seven independent operons as predicted by DOOR 2.0 operon analysis [37]. The ORFs within this cluster are predicted to encode a glycosyl transferase, glycosyl hydrolase, capsular polysaccharide biosynthesis/export periplasmic proteins, UDP-glucose lipid carrier transferase, and a mannose-1-phosphate guanylyl transferase/mannose-6-phosphate isomerase (S1 Table). We chose to study seven transposon insertional mutants (Bp1026b_I2907 (*becA*), Bp1026b_I2910 (*becD*), Bp1026b_I2914 (*becE*), Bp1026b_I2916 (*becG*), Bp1026b_I2921 (*becL*), Bp1026b_I2923 (*becN*), and Bp1026b_I2925 (*becP*)) from this polysaccharide gene cluster that exhibited reduced biofilm formation (Fig 1).

Interestingly, the transposon screen also identified four transposon insertional mutants in genes predicted to contribute to polysaccharide biosynthesis that are not associated with the exopolysaccharide biosynthetic gene cluster (Bp1026b_I2907-Bp1026b_I2927, *becA-R*). Two of these genes are Bp1026b_I0648, a glycosyl transferase family protein, and Bp1026b_I0649, a UDP-glucose 4-epimerase, both of which are part of a five gene cluster adjacent to the *wbiA* gene cluster responsible for lipopolysaccharide biosynthesis [51, 52]. The I0649::T24 mutant had the greatest reduction (>60%) in biofilm formation as compared to all of the other transposon insertional mutants in genes predicted to participate in polysaccharide biosynthesis (Fig 1). Additional transposon insertions that resulted in decreased biofilm formation (Fig 1) were identified in Bp1026b_II1959, a predicted glycosyltransferase that is part of the capsule III biosynthetic cluster [53], and Bp1026b_II2123, a predicted tyrosine-protein kinase.

### Bioinformatics analyses of predicted *B*. *pseudomallei* polysaccharide gene clusters

Exopolysaccharides are a key component of many bacterial biofilms [10], and the biofilm-associated exopolysaccharide biosynthetic gene cluster is conserved in other closely related species of the *B*. *pseudomallei* phylogenetic complex which includes *B*. *mallei* and *B*. *thailandensis* [54]. The exopolysaccharide biosynthesis genes are encoded by 18 loci and 3 pseudogene remnants spanning Bp1026b_I2907-Bp1026b_I2927 on chromosome I of *B*. *pseudomallei* 1026b and share high sequence identity with other clustered genes in *B*. *mallei* and *B*. *thailandensis* (S1 Fig and S3 Table). Nearly identical gene clusters were identified in the sequenced genomes of *B*. *mallei* ATCC 23344 and *B*. *thailandensis* E264 spanning the loci BMA0027-BMA0048 and BTH_I0520-BTH_I0537, respectively. Genetic alignments for *B*. *pseudomallei* and *B*. *mallei* revealed greater than 99% sequence identity at the nucleotide level or almost full conservation of this cluster. Alignments for *B*. *pseudomallei* and *B*. *thailandensis* identified an average sequence identity of 93.2% among the 18 loci, indicating a similarly high level of conservation. In particular, the gene locus predicted to encode for the mannose-1-phosphate guanylyltransferase, *manC* (Bp1026b_I2925, *becP*), shares 99.8% identity with BMA0029 in *B*. *mallei* and 94.1% identity with BTH_I0522 in *B*. *thailandensis*. These observations indicate a high degree of genetic conservation for this EPS cluster among closely related species despite differences in human and animal pathogenicity and environmental niche adaptation.

**Fig 2:**
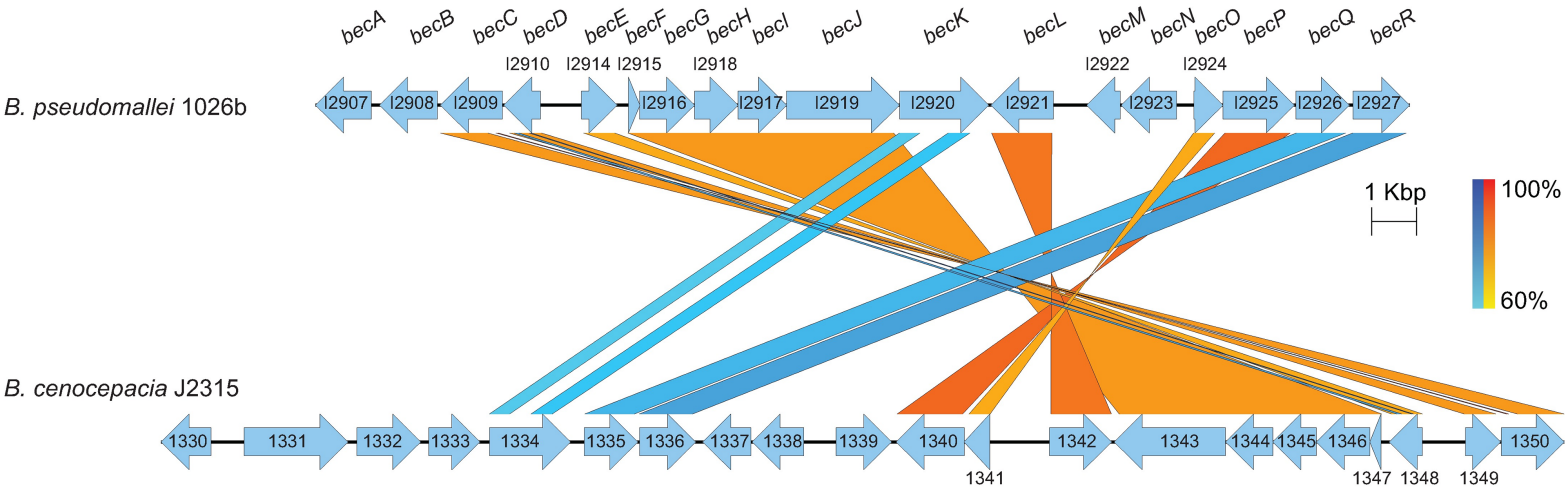
Biofilm-associated exopolysaccharide gene cluster from *B*. *pseudomallei* and comparative analysis with *B*. *cenocepacia*. The putative exopolysaccharide gene clusters from the sequenced genomes of *B*. *pseudomallei* 1026b (top) and *B*. *cenocepacia* J2315 (bottom). A total of 18 loci spanning Bp1026b_I2907-Bp1026b_I2927 (*becA*-*R*) on chromosome I of *B*. *pseudomallei* 1026b, not including a cluster of three pseudogenes, are aligned with 21 loci spanning BCAM1330-BCAM1350 on chromosome II of *B*. *cenocepacia* J2315. Coding sequences are depicted by arrows per positive or negative strand orientation. Sizes of genes and intergenic regions are to scale. The results of BLASTN annotations with minimum identity of 60% and threshold E-value of 1E-3 are aligned to regions of similarity. Red bars depict sequence inversions and blue bars depict direct homology in a color density gradient.

Beyond the closely related *B*. *pseudomallei* complex of bacteria, the *becA-R* biosynthetic cluster (Bp1026b_I2907-Bp1026b_I2927) was most highly conserved with a *B*. *cenocepacia* J2315 gene cluster (Fig 2 and S3 Table). Bioinformatics analyses comparing the putative exopolysaccharide gene clusters of *B*. *pseudomallei* 1026b and *B*. *cenocepacia* J2315 revealed high sequence conservation amid genetic rearrangement between the closely related pathogens. The exopolysaccharide cluster of *B*. *cenocepacia* J2315 has been previously reported to be encoded by loci BCAM1330-BCAM1341 on chromosome II and experimentally validated as a major structural component of biofilms [55]. We characterized the genetic sequence similarity based on the common ancestral origin of these strains in order to make comparisons with the biofilm-associated exopolysaccharide gene cluster identified from *B*. *pseudomallei* 1026b (Fig 2). Local pairwise alignments of genomic sequences using BLASTN showed high sequence homology within a region on chromosome I of *B*. *pseudomallei* 1026b and the cluster on chromosome II of *B*. *cenocepacia* J2315. Of the 18 predicted coding sequences that comprise the exopolysaccharide biosynthesis cluster, 14 are directly homologous to the exopolysaccharide cluster in *B*. *cenocepacia* J2315, spanning BCAM1334-BCAM1350 (Fig 2). The DNA sequences from 14 coding regions are 75.8% identical altogether and represent gene cluster homologues between the two *Burkholderia* species.

Interestingly, a majority of the homologous coding regions have flipped directional arrangements while maintaining high sequence identity (Fig 2). The coding regions of the *B*. *pseudomallei* 1026b cluster fully or almost fully align to homologous sequences in *B*. *cenocepacia* J2315 with percent identities ranging from 66–84% at the nucleotide level except for Bp1026b_I2920 (*becK*), which has an additional unique sequence in the middle of the gene interrupting alignment. A notable locus, Bp1026b_I2925 (*becP*), predicted to encode for mannose-1-phosphate guanylyltransferase (*manC*) that is required to catalyze the formation of nucleotide sugar GDP-mannose, shares 82.3% identity with BCAM1340. Additionally, Bp1026b_I2910 (*becD*), shares 74.9% identity with BCAM1349, the proposed transcriptional regulator of the exopolysaccharide gene cluster in *B*. *cenocepacia* J2315 [55].

These results indicate functional conservation of the exopolysaccharide cluster; however, our bioinformatics analysis revealed some crucial differences. Four loci in the 1026b predicted EPS cluster showed no homology to the J2315 cluster. The loci Bp1026b_I2907 (*becA*), I2908 (*becB*), I2922 (*becM*), and I2923 (*becN*), are predicted to encode a glycosyltransferase protein, a polysaccharide export periplasmic protein, a PAP2 superfamily protein, and a glycoside hydrolase family protein, respectively. Bp1026b_I2907 (*becA*) shares similarity to two predicted glycosyltransferases in the *B*. *cenocepacia* J2315 EPS cluster, BCAM1337 and BCAM1338 with 60.15% and 62.56% respective nucleotide identities. Likewise, Bp1026b_I2908 (*becB*) shares 61.89% nucleotide identity to BCAM1330, which is predicted to encode a putative polysaccharide export protein. However, the sequence correlations of Bp1026b_I2907 (*becA*) and Bp1026b_I2908 (*becB*) to *B*. *cenocepacia* J2315 do not pass our E-value threshold of 1e-3, representing a 0.001 chance of random sequence alignment, indicating that these correlations are not biologically significant. Bp1026b_I2922 (*becM*) and Bp1026b_I2923 (*becN*) also do not show significant sequence correlations to the *B*. *cenocepacia* J2315 genome; however, the flanking coding sequences of Bp1026b_I2921 (*becL*) and Bp1026b_I2924 (*becO*) appear homologous to BCAM1342 and BCAM1341, respectively, amid directional inversions (Fig 2). Interestingly, Bp1026b_I2921 (*becL*) and Bp1026b_I2924 (*becO*) are flanked by large noncoding intergenic regions totaling 1176bp, which co-localizes to an intergenic region spanning 1319bp in *B*. *cenocepacia* J2315. One explanation for this disparity involves the acquisition of the two coding sequences by *B*. *pseudomallei* 1026b for a species-specific fitness advantage.

Interestingly, we identified a transposon mutant insertion in Bp1026b_II1959, which is part of the capsule III biosynthetic cluster [53]. Our bioinformatics analysis revealed that this biosynthetic cluster (Bp1026b_II1956-Bp1026b_II1966) is homologous to the *bce-I* cluster that has been previously reported to synthesize the exopolysaccharide cepacian [40] in bacteria from the *Burkholderia cepacia* complex (S2A Fig and S4 Table). However, we only identified a single gene in this biosynthetic cluster in our biofilm screen and we did not identify any transposon insertion mutants in the *bce-II* cluster of the *Burkholderia cepacia* complex that shares strong homology with Bp1026b_II1796-Bp1026b_II1807 (S2B Fig and S4 Table).

### Swimming motility, growth rates, and colony morphology of biofilm-deficient mutants

**Fig 3:**
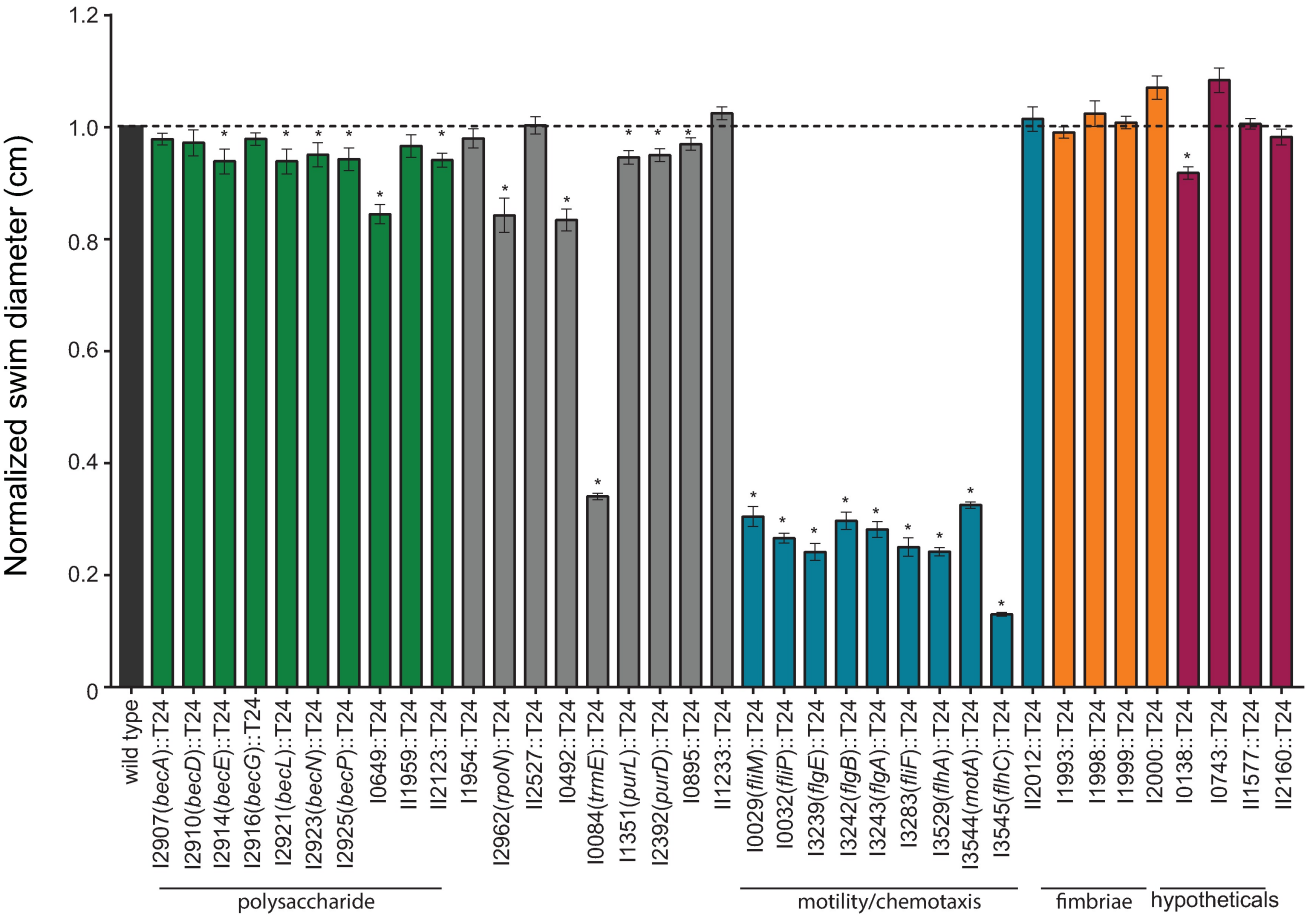
Swimming motility of *B*. *pseudomallei* T24 transposon mutants. Overnight cultures of the wild type and transposon mutants were used to inoculate 0.3% agar plates, incubated at 37°C, and swim zone diameter was measured at 24 h. Asterisks indicate a significant difference as obtained with the Mann-Whitney test utilizing the Bonferroni correction (p = 0.001) to account for multiple comparisons (n = 37). All mutants were tested at least twice in triplicate. Error bars indicate standard error of the mean.

Since motility can contribute to biofilm formation, we sought to address whether impaired motility contributed to decreased biofilm formation. A vast majority of the transposon mutants were not altered in swimming motility (Fig 3); however, not surprisingly, all transposon insertion mutants in genes involved in flagella production and assembly exhibited decreased motility (Fig 3). In addition, five other mutants (I0649::T24, I2962::T24 (*rpoN*), I0492::T24, I0084::T24 (*trmE*), I and 0138::T24) were minimally decreased in swim motility with the exception of the I0084::T24 (*trmE*) mutant, which was significantly impaired in swim motility (Fig 3).

In order to rule out the deleterious effect of potential growth defects on biofilm formation, we assayed all the strains used in these studies for rates of growth. A majority of the biofilm-defective mutants exhibited growth rates comparable to wild type when grown in LB medium for 48 h with the exception of I2962::T24 (*rpoN*), I0084::T24 (*trmE*), I1351::T24 (*purL*), I2392::T24 (*purD*), and II1233::T24 (S3 Fig).

**Fig 4:**
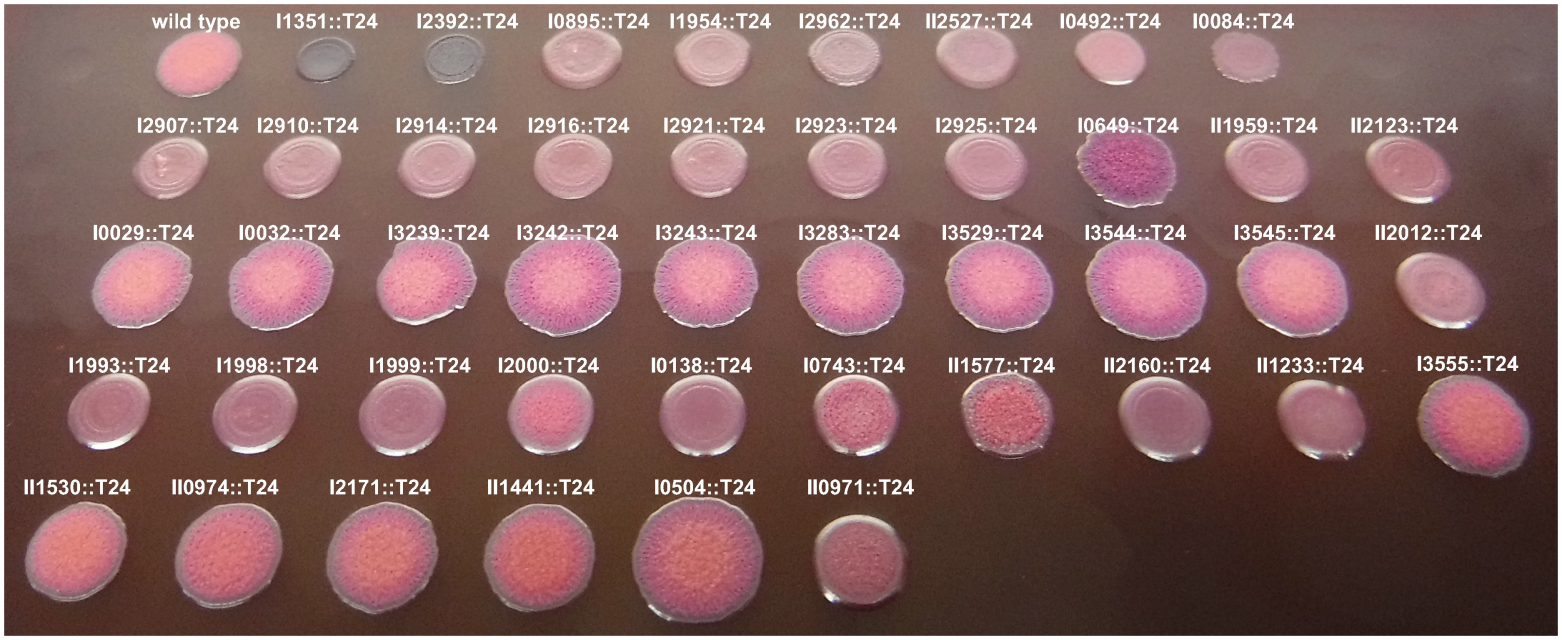
Growth of *B*. *pseudomallei* T24 transposon mutants on NAP-A plates. The wild type and transposon mutants were replica plated onto NAP-A agar plates using a pin replicator. Plates were incubated at 37°C for two days.

To provide an additional means to evaluate exopolysaccharide production, bacterial strains were cultivated on NAP-A agar which contains neutral red and crystal violet dyes [36]. Previous studies have reported the association of rugose colony morphology and the production of exopolysaccharides in a variety of Gram-negative bacteria [56–58]. The appearance of rugose (wrinkled) colony morphology and pellicle biofilm formation has also been reported to be linked to exopolysaccharide production in *B*. *cenocepacia* [55]. Thus, we hypothesized that the smooth appearance on NAP-A agar medium is directly or indirectly related to the loss or decreased production of exopolysaccharide. In this study, all of the transposon insertion mutants in the novel biofilm exopolysaccharide gene cluster (Bp1026b_I2907-Bp1026b_I2927, *becA-R*) and a majority of the remaining biofilm-deficient transposon mutants were smooth in appearance, while the motility mutants were rugose (Fig 4). Two transposon insertional mutants, I1351::T24 (*purL*) and I2392::T24 (*purD*), in genes involving purine biosynthesis appeared to preferentially uptake crystal violet and were smooth in appearance. Interestingly, one transposon mutant, I0649::T24 (a predicted UDP-glucose-4-epimerase), was more rugose (wrinkly) and heavily-pigmented as compared to wild type (Fig 4). A summary of the phenotypes for the biofilm-deficient transposon mutants can be found in Table 1.

**Table 1:** Summary of phenotypes (biofilm, motility, growth, and colony morphology) along with genetic annotations for selected biofilm-deficient transposon insertion mutants.

Locus	Gene	Biofilm (OD_600_)	Motility (cm)	Growth	Colony morphology	Annotation
wild type		1.25±0.03	4.07±0.05			
BP1026b_I0029	*fliM*	0.64±0.05	1.10±0.07	-	-	flagellar motor switch protein
BP1026b_I0032	*fliP*	0.46±0.03	0.96±0.03	-	-	flagellar biosynthesis protein
BP1026b_I0084	*trmE*	0.49±0.03	1.45±0.02	↓	smooth	tRNA modification GTPase
BP1026b_I0138		0.55±0.02	3.66±0.02	-	smooth	hypothetical protein
BP1026b_I0492		0.61±0.05	3.57±0.09	-	smooth	DnaA regulatory inactivator Hda
BP1026b_I0649		0.34±0.02	3.58±0.03	-	-, hyperpigmented	UDP-glucose 4-epimerase
BP1026b_I0743		0.38±0.01	4.31±0.06	-	-	hypothetical protein
BP1026b_I0895		0.57±0.02	4.13±0.08	-	smooth	sigma-54 interacting response regulator
BP1026b_I1351	*purL*	0.51±0.02	4.03±0.10	↓	smooth, purple	phosphoribosylformylglycinamidine synthase
BP1026b_I1954		0.17±0.01	4.20±0.12	-	smooth	DNA-binding response regulator
BP1026b_I1993		0.28±0.01	4.04±0.02	-	smooth	sensor histidine kinase/response regulator
BP1026b_I1998		0.32±0.02	3.86±0.02	-	smooth	hypothetical protein
BP1026b_I1999		0.29±0.01	4.01±0.04	-	smooth	hypothetical protein
BP1026b_I2000		0.45±0.02	4.13±0.07	-	smooth	lysR family transcriptional regulator
BP1026b_I2392	*purD*	0.58±0.03	4.05±0.09	↓	smooth, purple	phosphoribosylamine-glycine ligase
BP1026b_I2907	*becA*	0.52±0.04	4.24±0.05	-	smooth	glycosyl transferase group 1 protein
BP1026b_I2910	*becD*	0.45±0.03	4.14±0.07	-	smooth	hypothetical protein
BP1026b_I2914	*becE*	0.44±0.02	3.98±0.04	-	smooth	cyclic nucleotide-binding domain-containing protein
BP1026b_I2916	*becG*	0.84±0.08	4.28±0.09	-	smooth	acyl-CoA dehydrogenase domain-containing protein
BP1026b_I2921	*becL*	0.43±0.03	3.98±0.04	-	smooth	sigma-54 dependent transcriptional regulator
BP1026b_I2923	*becN*	0.44±0.03	4.03±0.05	-	smooth	glycoside hydrolase family protein
BP1026b_I2925	*becP*	0.66±0.02	4.00±0.04	-	smooth	mannose-1-phosphate guanylyltransferase /mannose-6-phosphate isomerase
BP1026b_I2962	*rpoN*	0.59±0.02	3.53±0.26	↓	smooth	RNA polymerase factor sigma-54
BP1026b_I3239	*flgE*	0.39±0.02	0.87±0.06	-	-	flagellar hook protein
BP1026b_I3242	*flgB*	0.42±0.02	1.07±0.06	-	-	flagellar basal body rod protein
BP1026b_I3243	*flgA*	0.57±0.02	1.01±0.05	-	-	flagellar basal body P-ring biosynthesis protein
BP1026b_I3283	*fliF*	0.38±0.03	0.90±0.06	-	-	flagellar MS-ring protein
BP1026b_I3529	*flhA*	0.38±0.02	0.87±0.03	-	-	flagellar biosynthesis protein
BP1026b_I3544	*motA*	0.56±0.02	1.17±0.02	-	-	flagellar motor protein
BP1026b_I3545	*flhC*	0.49±0.05	0.56±0.02	-	-	transcriptional activator
BP1026b_II1233		0.41±0.02	4.06±0.05	↓	smooth	transport/efflux protein
BP1026b_II1577		0.58±0.03	4.40±0.07	-	-	hypothetical protein
BP1026b_II1959		0.66±0.09	4.10±0.04	-	smooth	glycosyltransferase family protein
BP1026b_II2012		0.26±0.02	3.62±0.07	-	smooth	sensor histidine kinase/response regulator
BP1026b_II2123		0.49±0.01	4.00±0.09	-	smooth	exopolysaccharide tyrosine-protein kinase
BP1026b_II2160		0.33±0.01	4.30±0.10	-	smooth	hypothetical protein
BP1026b_II2527		0.52±0.03	4.30±0.12	-	smooth	sensor histidine kinase/response regulator

Minus symbol (-) indicates no difference from wild type.

### Complementation of I1954, I2907 (*becA*), and II2527 mutants

**Fig 5:**
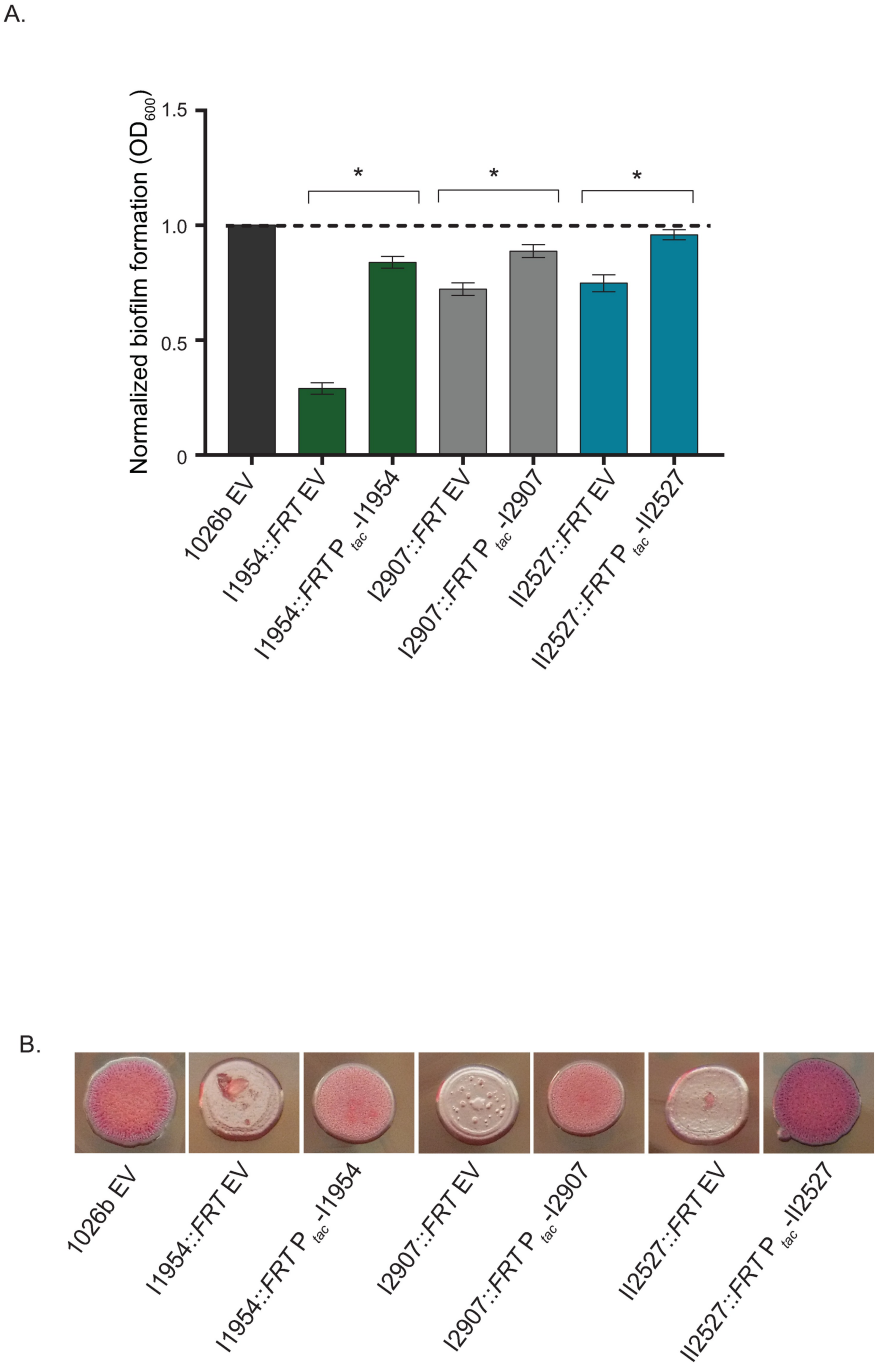
Complementation of I1954, I2907 (*becA*), and II2527 *FRT* mutants in the static biofilm assay and colony morphology on NAP-A agar plates. (A) Biofilm formation and (B) colony morphology of complemented I1954, I2907 (*becA*), and II2527 FRT mutants. EV indicates empty vector. Complementation was induced with 1mM IPTG. Asterisks indicate a significant difference as obtained with a pairwise Student’s t-test utilizing a p-value of 0.001. Error bars indicate standard error of the mean.

Complementation of representative mutant strains was achieved using a select-agent compliant methodology that removes the transposon leaving behind a *FRT* signature sequence, which still disrupts the reading frame of the targeted gene. The *FRT* mutants in a DNA response regulator (I1954::T24), a glycosyl transferase (I2907::T24 (*becA*)), and a sensor histidine kinase (II2527::T24) were complemented with the respective full length genes at a neutral Tn*7* site on the chromosome. Corresponding empty vector (EV) control strains were created for the wild type and the *FRT* mutants. Complementation of the I1954::*FRT* mutant expressing full length Bp1026b_I1954 significantly restored biofilm formation as compared to the I1954::*FRT* EV control strain (Fig 5A). It should be noted that I1954::T24 mutant insertional mutant exhibited the greatest decrease in biofilm formation from all mutants that were identified in the initial screen (Fig 1). The biofilm-defective phenotypes of the II2527::*FRT* and I2907::*FRT* mutants were also significantly complemented with full length clones as compared to their respective *FRT* EV control strains (Fig 5A). Additional evidence for complementation of all three *FRT* insertion mutants was also observed on NAP-A agar plates supplemented with IPTG. Wild type EV exhibited a rugose (wrinkly) phenotype on NAP-A, while all three *FRT* insertion mutants (I1954::*FRT*, I2907::*FRT* (*becA*), and II2527::*FRT*) exhibited a smooth phenotype that did not bind neutral red dye on these plates (Fig 5B). The rugose (wrinkly) phenotype was restored in all three transposon mutants when complemented with their respective full length genes (Fig 5B). Complementation of II2527::*FRT* not only restored the wrinkly phenotype, but the complemented clone also exhibited enhanced dye binding as compared to the wild type EV control and other *FRT* complemented strains (Fig 5B).

### Characterization of the biofilm-associated exopolysaccharide

**Fig 6:**
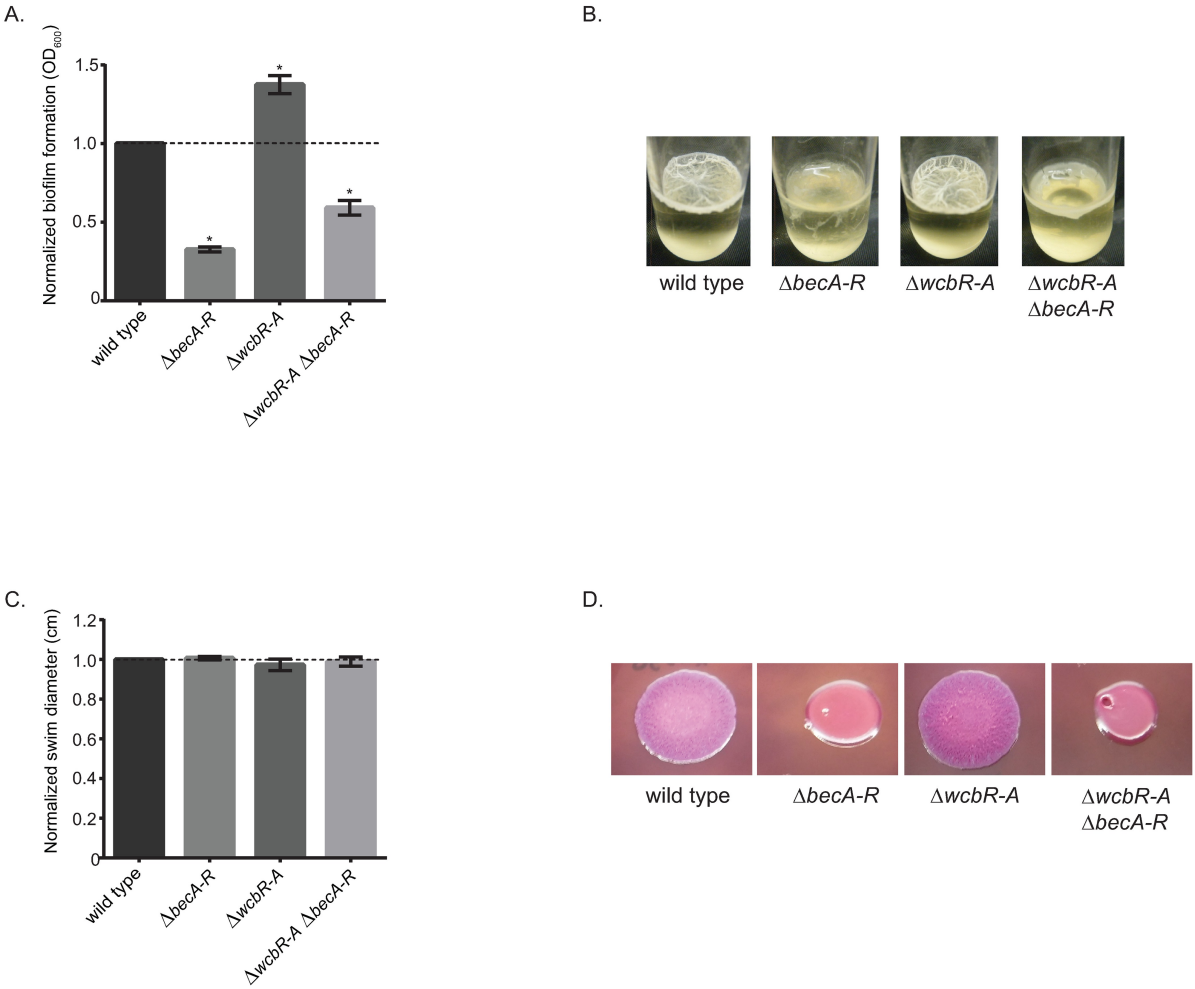
Deletion of biofilm exopolysaccharide gene cluster alters biofilm formation and growth on NAP-A plates, but not motility. (A) Biofilm formation of wild type, Δ*becA-R* (biofilm EPS deficient), Δ*wcbR-A* (CPSI deficient), and Δ*wcbR-A* Δ*becA-R* (CPSI and biofilm EPS deficient) strains after 24 h at 37°C. (B) Pellicle formation of wild type and deletion mutants after six days at 37°C. (C) Swim zone diameters (cm) of the wild type and deletion mutants after 24 h at 37°C. (D) Overnight cultures were spotted onto NAP-A plates and grown for two day at 37°C. Asterisks indicate a significant difference as obtained with a paired Student’s t-test utilizing for the biofilm data and the Mann-Whitney test for the swim motility data utilizing a p-value of 0.001. Error bars indicate standard error of the mean.

Given the importance of the exopolysaccharide biosynthetic gene cluster (Bp1026b_I2907-I2927) in biofilm formation, we have designated this gene cluster as *becA-R*. We generated a deletion mutant of the entire *becA-R* gene cluster (Bp1026b_I2907-I2927) in both the wild type and capsule I deficient (Δ*wcbR-A*) [44] backgrounds to further investigate how this exopolysaccharide contributes to *B*. *pseudomallei* biofilm formation. Deletion of the entire *becA-R* gene cluster in the wild-type background resulted in approximately a 63% decrease in biofilm formation (Fig 6A) and pellicle biofilm formation was also impaired (Fig 6B). Interestingly, deletion of capsule I resulted in a 40% increase in biofilm formation which is consistent with a previously published report [53] (Fig 6A). Loss of both capsule I (Δ*wcbR-A*) and the biofilm-associated exopolysaccharide (Δ*becA-R*) reduced the biofilm 40%, which was an intermediate phenotype as compared to the wild type and the exopolysaccharide deletion mutant (Fig 6A). Motility was not significantly altered in either the Δ*wcbR-A*, Δ*becA-R* or the Δ*wcbR-A* Δ*becA-R* double mutants as compared to wild type (Fig 6C). Interestingly, the wild type and the Δ*wcbR-A* mutant exhibited rugose colony morphology in contrast to the Δ*becA-R* mutant and the Δ*wcbR-A* Δ*becA-R* double mutant that were smooth in appearance on NAP-A agar (Fig 6D). Rugose colony morphology is often associated with the production of exopolysaccharides [55–58].

**Fig 7:**
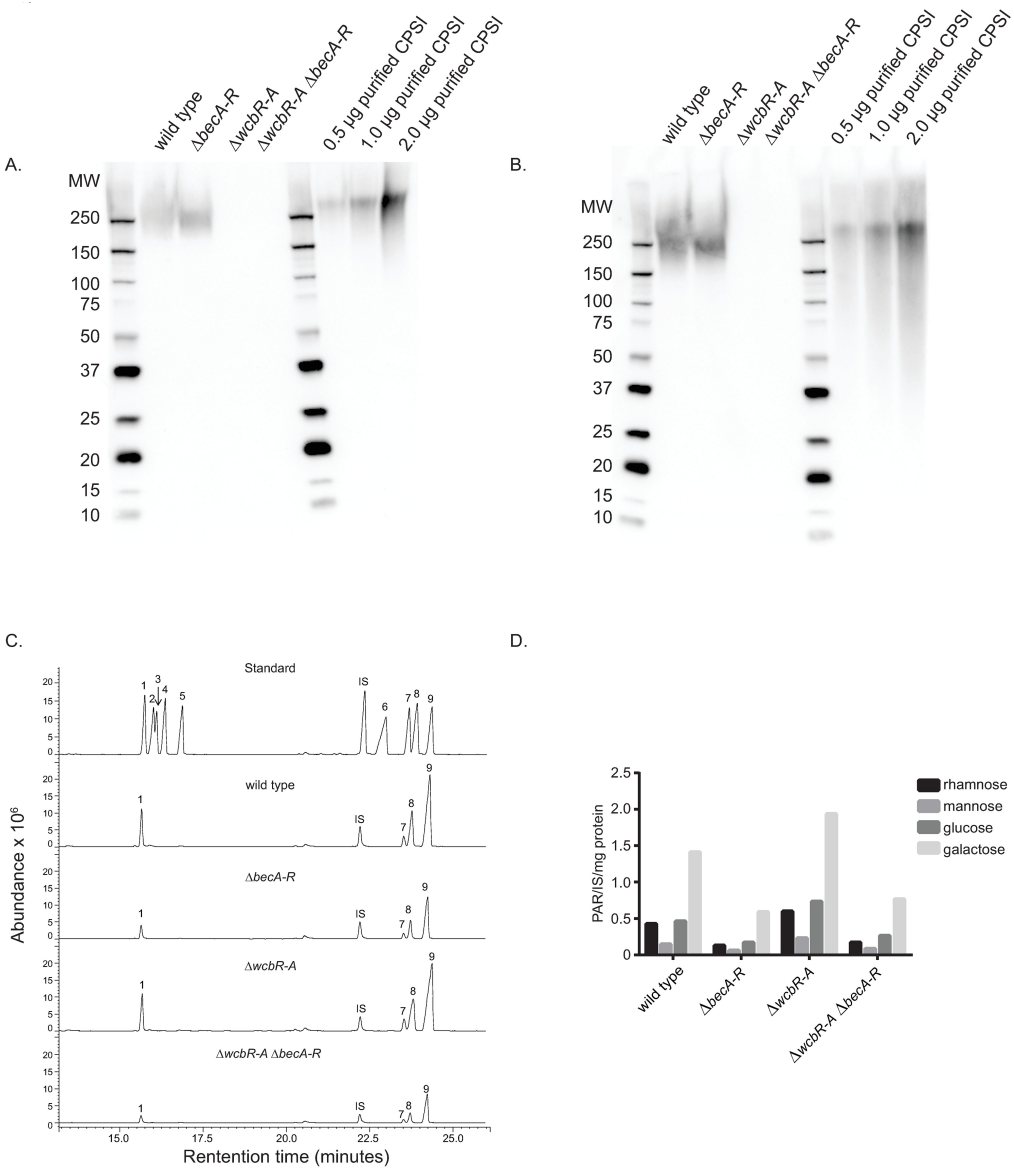
Western blot and GC-MS of the exopolysaccharide deletion mutant. Detection of polysaccharides with mAb 3015 (A) or the CPSI capsular polysaccharide-specific mAb 4C4 (B) from wild type (Bp82), Δ*becA-R* (biofilm EPS deficient), Δ*wcbR-A* (CPSI deficient), Δ*wcbR-A* Δ*becA-R* (CPSI and biofilm EPS deficient) preparations, and purified CPSI (0.5, 1.0, and 2.0 μg) from Bp82. (C) Typical GC-MS total ion chromatograms of monosaccharides (standards peaks: 1-rhamnose, 2-ribose, 3-fucose, 4-arabinose, 5-xylose, 6-inositol, 7-mannose, 8-glucose, and 9-galactose. IS = internal standard (3-O-methyl-glucose)). (D) Relative abundance of sugars from Bp82 exopolysaccharide extracts. Each monosaccharide was normalized with an internal standard (PAR = peak area ratio) and normalized against sample protein content.

We conducted western blot analysis on polysaccharide preparations in order to differentiate the biofilm-associated exopolysaccharide encoded by *becA-R* from the previously described capsular polysaccharide (CPSI) and >150kDa acidic exopolysaccharide from *B*. *pseudomallei* [13–15, 59]. Western blot analysis on polysaccharide preparations from Δ*becA-R* and Δ*wcbR-A* Δ*becA-R* double mutants indicated that the biofilm-associated exopolysaccharide described in the current study and the previously described acidic exopolysaccharide are not the same using an antibody (mAb 3015) raised against the acidic exopolysaccharide (Fig 7A). Cross reactivity with purified CPSI suggested that the acidic exopolysaccharide-specific antibody reacts with a constituent of CPSI (Fig 7A). We also confirmed that the polysaccharide preparations produced by the *becA-R* biosynthetic cluster were not CPSI (Fig 7B), since the purified CPSI [14] and preparations from Δ*becA-R* are reactive to the CPSI-specific 4C4 IgG1 antibody [46, 60, 61], whereas the preparations from Δ*wcbR-A* mutant and the Δ*wcbR-A* Δ*becA-R* double mutants are not reactive to the CPSI-specific 4C4 IgG1 antibody.

Carbohydrate analysis of polysaccharide preparations from wild-type *B*. *pseudomallei* Bp82 indicated that these preparations are comprised primarily of four monosaccharides: glucose, galactose, rhamnose and mannose, in a ratio of 0.46:1.41:0.43:0.14, respectively (Fig 7C and 7D). Comparative analysis suggested that there is a decrease of all four monosaccharides, rhamnose, mannose, glucose, and galactose in the Δ*becA-R* or Δ*wcbR-A* Δ*becA-R* double mutant as compared to the wild-type Bp82 (Fig 7D).

### Previously published genes that contribute to *B*. *pseudomallei* biofilm formation

**Fig 8:**
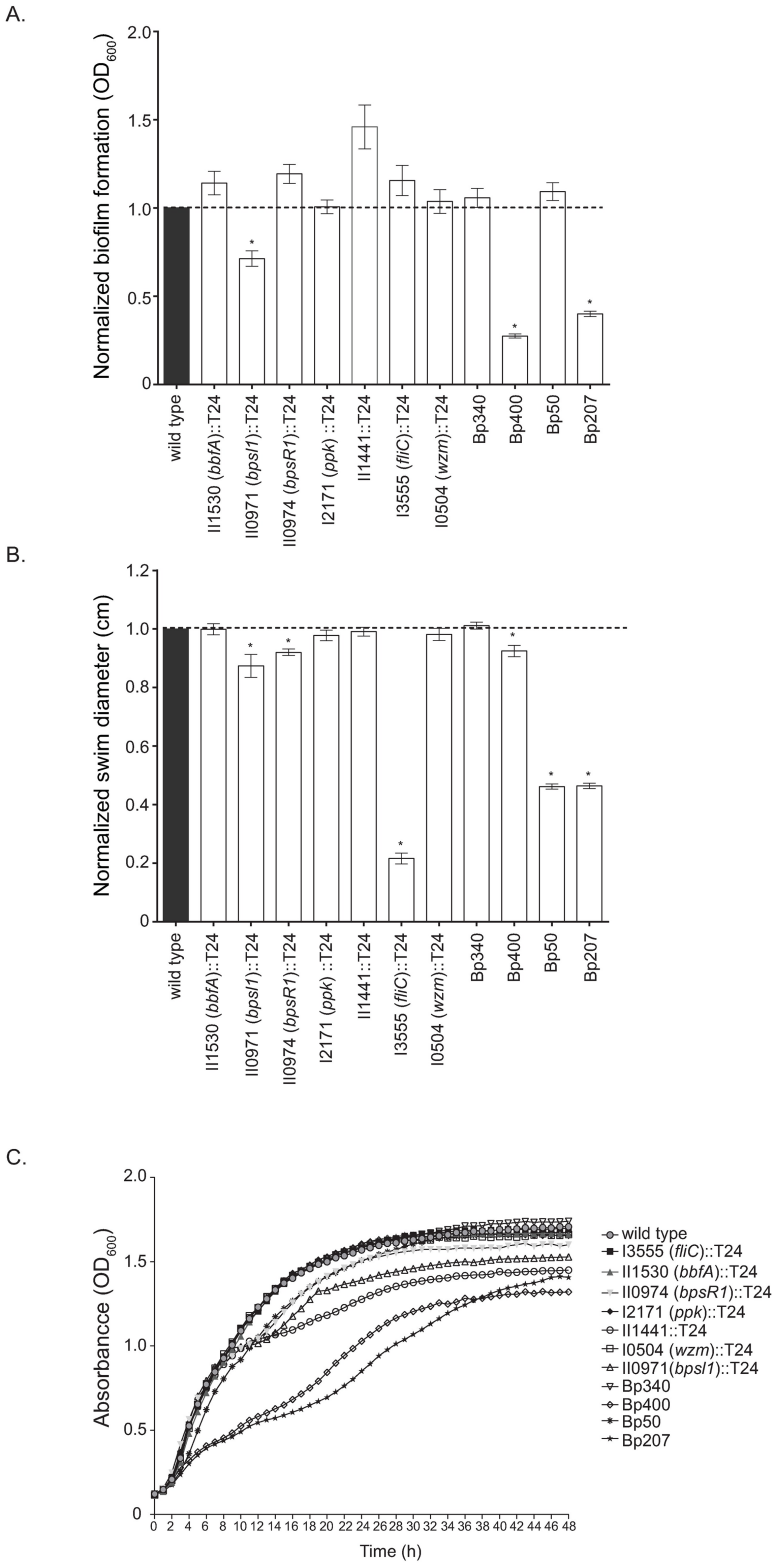
Biofilm formation, swimming, and growth of published biofilm *B*. *pseudomallei* mutants. (A) Biofilm formation of the wild type and T24 transposon mutants in genes previously reported to be involved in biofilm production and four efflux pump deletion mutants grown at 37°C for 24 h. (B) The wild type and transposon/deletion mutants were inoculated into 0.3% agar plates, incubated at 37°C for 24 h, and diameters measured. (C) Growth curves of transposon/deletion mutants over 48 h at 37°C. Asterisks indicate a significant difference as obtained with a paired Student’s t-test for the biofilm data and the Mann-Whitney test for the swim motility data utilizing a p-value of 0.001. Error bars indicate standard error of the mean.

In addition to characterizing the biofilm-defective mutants identified in our screen, we sought to characterize seven transposon insertion mutants and four deletion mutants in *B*. *pseudomallei* genes previously described to contribute to biofilm formation in the literature (S5 Table), since these were candidate genes that we expected to identify in our screen [19–24, 62]. Seven transposon insertional mutants from the *B*. *pseudomallei* 1026b T24 library in addition to four efflux pump deletion mutants were tested in various assays (S5 Table). Under the conditions used in our screen, only one of the seven transposon mutants, II0971::T24 (*bpsl1*), recapitulated a reduced biofilm phenotype that had been previously reported [18] and two (Bp400 Δ*bpeAB*-*oprB*::*FRT* Δ*amrRAB*-*oprA* and Bp207 Δ*amrRAB*-*oprA*::*FRT* Δ*bpeAB*-*oprB*::*FRT*) of the four efflux pump deletion mutants exhibited a significant reduction in biofilm formation (Fig 8A and Table 2). However, the initial pellicle biofilm screen did identify transposon insertion mutants in a two-component regulator, *bfmR* (Bp1026b_I1992), and the corresponding pili biosynthesis genes that it regulates [21].

**Table 2:** Summary of phenotypes (biofilm, motility, growth, and colony morphology) for selected transposon insertion and deletion mutants in previously published genes that contribute to biofilm formation.

Locus	Gene	Biofilm (OD_600_)	Motility (cm)	Growth	Colony morphology
I3555::T24	*fliC*	1.22±0.07	0.83±0.06	-	-
II1530::T24	*bbfA*	1.16±0.06	3.83±0.05	-	-
II0974::T24	*bpsR1*	1.44±0.04	3.53±0.03	↓	-
I2171::T24	*ppk*	1.23±0.04	3.77±0.04	-	-
II1441::T24		1.72±0.10	3.80±0.04	↓	-
I0504::T24	*wzm*	1.16±0.05	3.76±0.05	↓	-
II0971::T24	*bpsl1*	0.87±0.08	3.45±0.04	↓	-
Bp340		1.21±0.05	3.88±0.04	-	-
Bp400		0.32±0.01	3.55±0.08	↓	-
Bp50	*purL*	1.26±0.05	1.77±0.03	-	-
Bp207		0.46±0.02	1.78±0.03	↓	-
I3555::T24		1.22±0.07	0.83±0.06	-	-
II1530::T24		1.16±0.06	3.83±0.05	-	-
II0974::T24		1.44±0.04	3.53±0.03	↓	-
I2171::T24		1.23±0.04	3.77±0.04	-	-

Minus symbol (-) indicates no difference from wild type.

We also evaluated whether these mutants were altered in swim zone diameter or growth rate under our conditions. Two transposon insertional mutants in quorum sensing, II0971::T24 (*bpsl1*) and II0974::T24 (*bpsR1*), were impaired in swim zone diameter (Fig 8B), while one transposon mutant, I3555::T24 (*fliC*), was significantly impaired in swim zone diameter (Fig 8B). The swim zone diameters of two efflux pump mutants (Bp50 Δ*amrRAB*-*oprA* and Bp207 Δ*amrRAB*-*oprA* Δ*bpeAB*-*oprB*) were reduced by roughly 50% (Fig 8B). Two transposon mutants, II1441::T24 and II0971::T24 (*bpsl1*) in addition to two efflux pump mutants, Bp207 Δ*amrRAB*-*oprA* Δ*bpeAB*-*oprB* and Bp400 Δ*amrRAB*-*oprA* Δ*bpeAB*-*oprB* exhibited delayed or slowed growth over 48 h as compared to wild type (Fig 8C). The transposon insertional mutants in genes previously described to be biofilm defective exhibited a rugose appearance similar to wild type on NAP-A agar (Fig 4). However, the colony morphology of the efflux pump mutants was not fully assessed due to the sensitivity of these mutants to the antibiotics in the NAP-A medium. A summary of the phenotypes for the previously published biofilm mutants can be found in Table 2.

## Discussion

*B*. *pseudomallei* is the etiological agent of melioidosis, a disease that is often misdiagnosed due to its many clinical manifestations. Inaccurate or delayed diagnoses, lack of a vaccine, evasion of the immune system, and intrinsic antibiotic resistance contribute to the high mortality rate of this disease. Although, the precise role(s) of *B*. *pseudomallei* biofilm formation in the initiation of an infection and continued persistence in a mammalian host is not fully understood, the identification of the genes that contribute to biofilm formation may provide some insight to address these fundamental questions. It has been previously described that *B*. *pseudomallei* can be found in unusual locations in the human body and produce exopolysaccharides that contribute to the evasion of phagocytosis and persistence of chronic infections [63]. A better understanding of the tolerance associated with *B*. *pseudomallei* biofilms to antibiotics may help to explain the lack of success in the treatment of the chronic manifestations of melioidosis [64]. In addition, clinical studies suggest that melioidosis relapse may be associated with the biofilm-forming capacity of the primary infecting isolate [65].

To gain a better understanding of the genes that contribute to *B*. *pseudomallei* biofilm formation, we utilized a sequence-defined two allele transposon library of *B*. *pseudomallei* 1026b to identify 59 genetic loci involved in biofilm formation. This functional based screening approach identified non-essential genes that are directly associated with biofilm formation. The additional phenotypic characterization of these transposon insertional mutants (Table 1) allows for a more detailed analysis of the contribution of these genes to biofilm formation, as opposed to alternative analysis methods that rely on global transcriptional profiling of non-isogenic strains or strains that have an evolutionary relationship to *B*. *pseudomallei*. However, these approaches complement the results of the studies reported here. A recent transcriptome analysis of two non-isogenic strains of *B*. *pseudomallei* that produce high and low levels of biofilm identified 563 differentially regulated genes using RNA-seq that may contribute to biofilm formation [66]. In this study, we identified and characterized some of the same genes using a functional-based screening approach. Additionally, another RNA-seq based study of a contact-dependent growth inhibition system [67] and the corresponding genes regulated from *B*. *thailandensis* identified genes that are homologous to the biofilm exopolysaccharide biosynthesis, fimbriae production, and an exopolysaccharide tyrosine-protein kinase identified in this study.

A subset of the genes identified in this study has previously been highlighted in other research studies (Table 2). The sensor histidine kinase encoded by Bp1026b_I1993 has been previously shown to be up-regulated during an acute infection in Syrian hamsters and a deletion mutant exhibited a higher LD_50_ as compared to the wild type suggesting a potential role as a virulence factor during infection [68]. The ability to attach to host tissues is a crucial step during infection and biofilm formation. More recently, seven out of the nine genes in this fimbriae gene cluster were reported to be differentially regulated between low and high biofilm producing clinical isolates [66]. In addition, an insertion mutant of BPSL2024 (Bp1026b_I1992), designated as *bfmR* (biofilm formation associated regulator), which is a predicted DNA-binding response regulator, exhibited poor growth under iron-limiting conditions, reduced biofilm-forming capacity (~70%), and reduced fimbriae production, while motility was comparable to the wild type [21]. In this study, we phenotypically characterized four mutants in this fimbriae gene cluster, I1993::T24, I1998::T24, I1999::T24, and I2000::T24, although we had initially identified all nine genes in this gene cluster for their contribution to biofilm formation.

Conflicting reports on the role of biofilm components has further complicated our current ability to determine the role of biofilms in melioidosis [25–27]. As a means to compare our results with the previously published body of literature, we evaluated transposon mutants in the genes previously described to contribute to biofilm formation. A majority of the transposon mutants in published genes with a reported biofilm deficient phenotype that we retested from our library did not exhibit a decrease in biofilm formation under the conditions tested. This could be attributed to variation in the conditions tested, the genetic background of the strains, or the method of gene inactivation used in those studies.

A primary example of a potential discrepancy as observed in this study is the transposon insertion in *fliC*. Under the conditions used in this study, I3555::T24 (*fliC*) was defective in swimming motility, but still competent to form biofilms (Table 2). We also previously characterized that this transposon insertion mutant did not produce detectable levels of FliC in western blot analyses [35]. Interestingly, other transposon insertion mutations in genes that contribute to flagella biogenesis and function (e.g. *fliM*, *fliP*, *flgE*, *flgB*, *fliF*, *motA*, *flhA*, and *flhC*) were identified to be defective in both motility and biofilm formation in this study. These results suggest that flagella biogenesis and function contributes to pellicle biofilm formation and static biofilm formation under the conditions tested; however, it is presently unclear as to the exact role of the FliC filament in biofilm formation. Biofilm studies have previously evaluated the role of *fliC* in an aflagellate transposon insertion mutant designated as MM35 under a variety of conditions in various biofilm formation assays, which resulted in an approximate 40 to 77% reduction in biofilm forming capacity in these published reports [22, 62]. The variability of the MM35 *fliC* mutant as reported by those previous studies is an indication that contribution of FliC is conditionally dependent and other factors may share a functionally redundant role with other factors to promote biofilm formation. Future efforts will be directed at further defining the role of FliC under various conditions.

Due to the multitude of factors that contribute to biofilm formation and the nature of our initial screen (qualitative pellicle biofilm assay), there are potentially more genes that are involved in biofilm formation that were not identified in our screen. These genes may be detected in more quantitative assays when screened under more diverse growth conditions (temperature, carbon source, oxygen tension, etc.). As of yet, the role of biofilm formation and biofilm-specific components has yet to be elucidated [19]. One of the major issues associated with the conclusions that have been made so far is the lack of studies that look at the role of biofilms in chronic infection models of melioidosis. To address important research questions in a model *B*. *pseudomallei* strain as a community, we have validated this sequence-defined transposon insertion library used in this study and deposited it with BEI resources.

Deletion of the entire *B*. *pseudomallei* biofilm exopolysaccharide cluster led to a significant decrease in biofilm formation and smooth appearance on NAP-A (Fig 6A and 6D), which is indicative of the loss of exopolysaccharide production. Further investigation by Western blot analysis with mAbs specific for the CPSI and the previously described acidic polysaccharide indicated that the product of the *becA-R* biosynthetic cluster does not contribute to the production of previously characterized polysaccharides identified by these antibodies. The analysis of the CPSI-deficient mutant (*wcbR-A*) and purified CPSI capsular polysaccharide ([→3)-2-O-acetyl-6-deoxy-β-D-*manno*-heptopyranose-(1→]) also indicated that mAb 3015 detects a polysaccharide component that is produced by the genes previously characterized to contribute to CPSI biosynthesis and may likely be specific to CPSI. One might speculate that the previously reported reactivity of the acidic polysaccharide antibody [59] was potentially due to minor CPSI contamination during the acidic exopolysaccharide extraction. Alternatively, this antibody might be cross-reactive with both polysaccharides and deletion of the CPSI gene cluster might also effect the expression of the acidic exopolysaccharide.

The role of CPSI in biofilm formation was also highlighted in our epistatic analysis of polysaccharide biosynthesis clusters. In our study, the deletion of CPSI biosynthesis genes increases biofilm formation, which has been previously observed [53]. This may indicate that CPSI production alters the dynamics of biofilm attachment and subsequent formation. These effects could also be the result of altered cellular levels of nucleotide sugar precursors used in polysaccharide biosynthesis. Interestingly, biofilm formation is also increased in the Δ*wcbR-A* Δ*becA-R* double mutant as compared to Δ*becA-R*, indicating that capsule production effects biofilm formation in the absence of the biofilm-associated exopolysaccharide. Thus, CPSI has an overriding contribution and generates interesting complications for the study of *B*. *pseudomallei* biofilm formation. Future biofilm studies that aim to understand the biofilm physiology and dynamics of *B*. *pseudomallei* biofilm growth will have to be performed in strains that produce CPSI based on the effects that this EPS component has on biofilm formation. Excluding CPSI from future biofilm analyses conducted in closely related *Burkholderia* species or strains that do not produce CPSI will confound the elucidation of the role of biofilms in melioidosis.

We determined through carbohydrate analysis that the biofilm-associated exopolysaccharide is comprised of four primary monosaccharides: glucose, galactose, rhamnose, and mannose, which is consistent with a recent report on a biofilm-associated exopolysaccharide purified from *B*. *pseudomallei* [69]. However, the ratio of the four monosaccharides reported differs between our two studies, which may be reflective of differences in growth conditions, method of polysaccharide purification, or strains under investigation. It is also unclear if the exopolysaccharide evaluated previously [69] is the sole product of the *becA-R* cluster that we have characterized in this study.

As *Burkholderia* species are known to produce a diversity of exopolysaccharides, we conducted additional bioinformatics analyses to further characterize the biofilm-associated exopolysaccharide. Based on our genomics analyses, the *becA-R* cluster reported here is not part of the evolutionarily conserved cepacian biosynthesis clusters (*bce-I* and *bce-II*). However, additional exopolysaccharide biosynthetic clusters, which includes the cepacian cluster, are present in other locations within the *B*. *pseudomallei* genome and have yet to be fully characterized. Interestingly, the *becA-R* is highly conserved between *B*. *pseudomallei*, *B*. *mallei* and *B*. *thailandensis*. The strong conservation of the *becA-R* cluster during *B*. *pseudomallei* genome adaptation and reduction in *B*. *mallei* suggests that the biofilm-associated cluster contributes to pathogenesis, as genes that are not necessary for living in an animal host would have been predicted to be lost during genome reduction [70]. This is in contrast to the *bce-I* cluster (*B*. *cepacia* complex annotation) that has been lost in *B*. *mallei* [40], which is often referred to as capsule III in *B*. *pseudomallei*. Together, these results suggest an evolutionary relationship and differentiation of functional roles for the EPS components in *Burkholderia* species and a critical need to understand their role in biofilm formation and pathogenesis.

The literature continues to expand and modify the structural characterization of surface-associated polysaccharides in *B*. *pseudomallei*. However, a critical need exists to link the polysaccharides produced and their corresponding biosynthetic genes. Additional research will be conducted to determine the precise composition and structure of the *B*. *pseudomallei* biofilm exopolysaccharide produced by *becA-R*. Additional studies will also be geared at generating monoclonal antibodies for future diagnostics efforts. The ability of bacterial pathogens to attach, colonize surfaces, and form a biofilm is a key first step in the initiation of pathogenesis and evasion of host defenses. In this paper, we identified and characterized the genetic loci that contribute to *B*. *pseudomallei* biofilm formation. We also identified an exopolysaccharide that is essential for biofilm formation, which is confined to a few closely related *Burkholderia* species that comprise the *B*. *pseudomallei* complex. To date, the majority of published studies on *B*. *pseudomallei* EPS have focused on capsular polysaccharide I (CPSI). However, the *B*. *pseudomallei* genome encodes the capacity for expression of multiple additional capsular polysaccharides and secreted exopolysaccharides. The nature and role of these additional EPS components remains to be characterized in the context of *B*. *pseudomallei* tissue tropism, biofilm formation, antibiotic tolerance, and persistence in the host. Our future efforts will be focused on characterizing the growth of *B*. *pseudomallei* as a biofilm to understand how biofilm growth contributes to antimicrobial tolerance and the failure of antibiotic treatment in patients with melioidosis.

## Supporting information

S1 Fig Comparative analysis of *becA-R* biofilm gene cluster from *B*. *mallei* ATCC23344, *B*. *pseudomallei* 1026b, and *B*. *thailandensis* E264. The *becA-R* gene cluster from the sequenced genomes of *B*. *mallei* ATCC23344 (top), *B*. *pseudomallei* 1026b (middle), and *B*. *thailandensis* E264 (bottom). Genes for *becA-R* of *B*. *pseudomallei* 1026b, Bp1026b_I2907-Bp1026b_I2927 (*becA*-*R*) are aligned with BMA0027-BMA0048 from *B*. *mallei* ATCC and *B*. *thailandensis* E264 BTH_I0520-BTH_I0537. Coding sequences are depicted by arrows per positive or negative strand orientation and sizes of genes and intergenic regions are to scale. The results of BLASTN annotations with minimum identity of 60% and threshold E-value of 1E-3 are aligned to regions of similarity. Red bars depict sequence inversions and blue bars depict direct homology in a color density gradient.
(TIF)

S2 Fig Comparative analysis of *bce-I* and *bce-II* gene clusters from *B*. *pseudomallei* and *B*. *vietnamiensis* G4. The cepacian biosynthesis (*bce-I* and *bce-II*) gene clusters from the sequenced genomes of *B*. *pseudomallei* 1026b (top) and *B*. *vietnamiensis* G4 (bottom). (A) Genes for *bce-I* of *B*. *pseudomallei* 1026b, Bp1026b_II1966-Bp1026b_II1956 are aligned with Bcep1808_4200-Bcep1808_4210 from *B*. *vietnamiensis* G4. (B) Genes for *bce-II* of *B*. *pseudomallei* 1026b, Bp1026b_II1796-Bp1026b_II1807 are aligned with Bcep1808_4471-Bcep1808_4480 from *B*. *vietnamiensis* G4. Coding sequences are depicted by arrows per positive or negative strand orientation and sizes of genes and intergenic regions are to scale. The results of BLASTN annotations with minimum identity of 60% and threshold E-value of 1E-3 are aligned to regions of similarity. Red bars depict sequence inversions and blue bars depict direct homology in a color density gradient.
(TIF)

S3 Fig Growth curves of *B*. *pseudomallei* 1026b biofilm mutants. Overnight cultures were grown in LB and cultures were adjusted to a final OD_600_ 0.1. Bacteria were grown at 37°C with shaking. Readings were taken every hour (A-D).
(TIF)

S1 Table Biofilm-defective transposon mutants identified in primary screen. Columns presented are the old NCBI gene locus, new NCBI gene locus, K96243 gene locus, gene description as found in burkholderia.com [38], gene locus, and if the gene was noted to have increased gene expression in a recent transcriptomic study [66]. Gene loci in bold represent transposon mutants that are predicted to be the first gene in an operon and were studied in detail.
(TIF)

S2 Table Strains and plasmids used in the study. (TIF)

S3 Table Percent nucleotide and amino acid identities of the *becA-R* biofilm exopolysaccharide gene clusters from *B*. *pseudomallei* 1026b as compared to *B*. *mallei* ATCC2334, *B*. *thailandensis* E264, and *B*. *cenocepacia* J2315 exopolysaccharide gene clusters. (TIF)

S4 Table Percent nucleotide identity of the *bce-I* and *bce-II* gene clusters from *B*. *pseudomallei* 1026b compared to *B*. *vietnamiensis* G4, *B*. *cenocepacia* J2315, *B*. *thailandensis* E264, and *B*. *mallei* ATCC23344. (TIF)

S5 Table Published *B*. *pseudomallei* genes that contribute to biofilm formation. The asterisk indicates that a transposon insertional mutant was identified in the screen described in the current study.
(TIF)
