# Supplementary figures and images for: Genome-scale analysis of the genes that contribute to Burkholderia pseudomallei biofilm formation identifies a crucial exopolysaccharide biosynthesis gene cluster
Source: PLoS Negl Trop Dis. 2017 Jun 28;11(6):e0005689. doi: 10.1371/journal.pntd.0005689 (PMC5507470; doi:10.1371/journal.pntd.0005689)

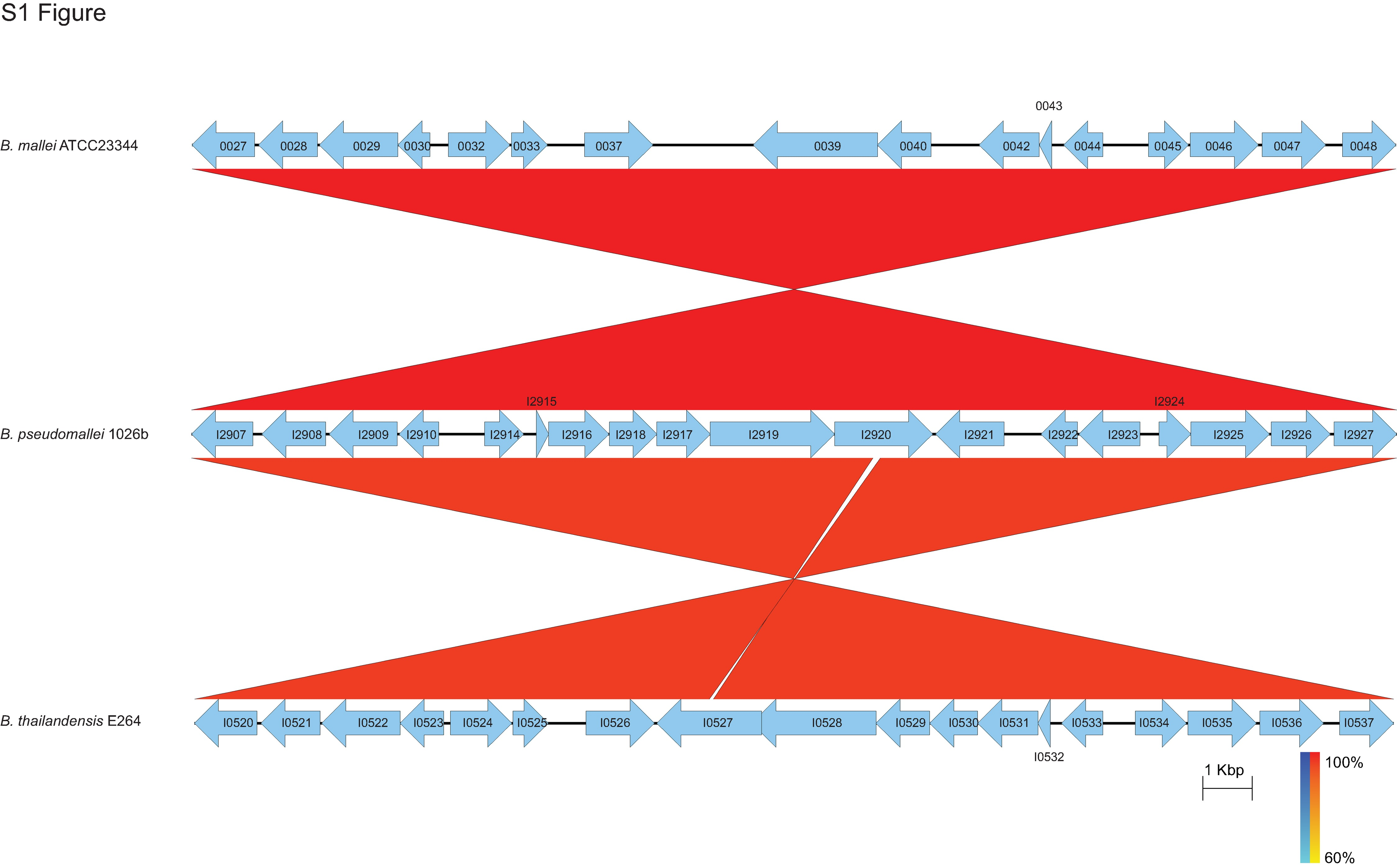

Supplement: S1 Fig — The becA-R gene cluster from the sequenced genomes of B. mallei ATCC23344 (top), B. pseudomallei 1026b (middle), and B. thailandensis E264 (bottom). Genes for becA-R of B. pseudomallei 1026b, Bp1026b_I2907-Bp1026b_I2927 (becA-R) are aligned with BMA0027-BMA0048 from B. mallei ATCC and B. thailandensis E264 BTH_I0520-BTH_I0537. Coding sequences are depicted by arrows per positive or negative strand orientation and sizes of genes and intergenic regions are to scale. The results of BLASTN annotations with minimum identity of 60% and threshold E-value of 1E-3 are aligned to regions of similarity. Red bars depict sequence inversions and blue bars depict direct homology in a color density gradient. (TIF) [file pntd.0005689.s001.tif]

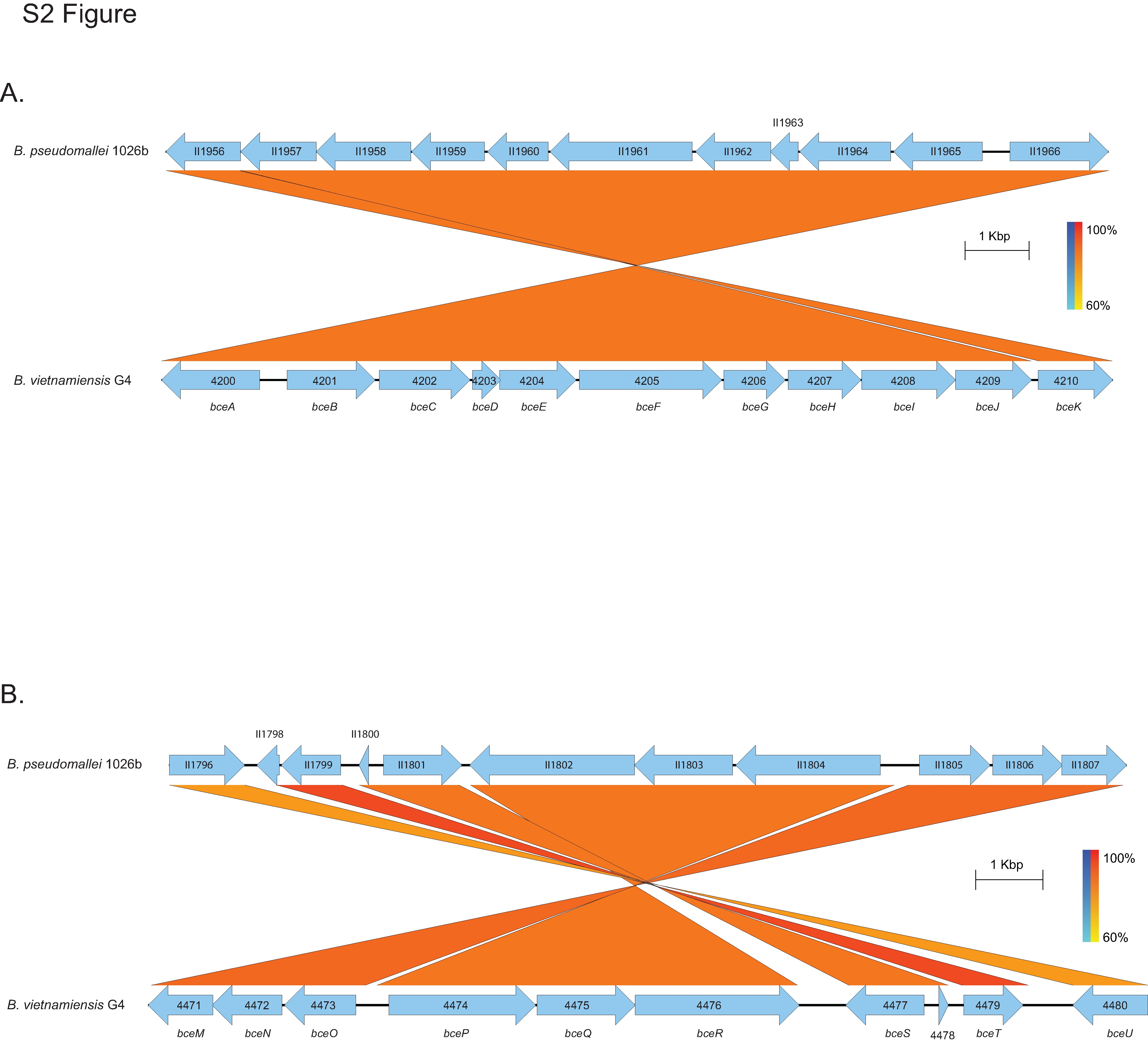

Supplement: S2 Fig — The cepacian biosynthesis (bce-I and bce-II) gene clusters from the sequenced genomes of B. pseudomallei 1026b (top) and B. vietnamiensis G4 (bottom). (A) Genes for bce-I of B. pseudomallei 1026b, Bp1026b_II1966-Bp1026b_II1956 are aligned with Bcep1808_4200-Bcep1808_4210 from B. vietnamiensis G4. (B) Genes for bce-II of B. pseudomallei 1026b, Bp1026b_II1796-Bp1026b_II1807 are aligned with Bcep1808_4471-Bcep1808_4480 from B. vietnamiensis G4. Coding sequences are depicted by arrows per positive or negative strand orientation and sizes of genes and intergenic regions are to scale. The results of BLASTN annotations with minimum identity of 60% and threshold E-value of 1E-3 are aligned to regions of similarity. Red bars depict sequence inversions and blue bars depict direct homology in a color density gradient. (TIF) [file pntd.0005689.s002.tif]

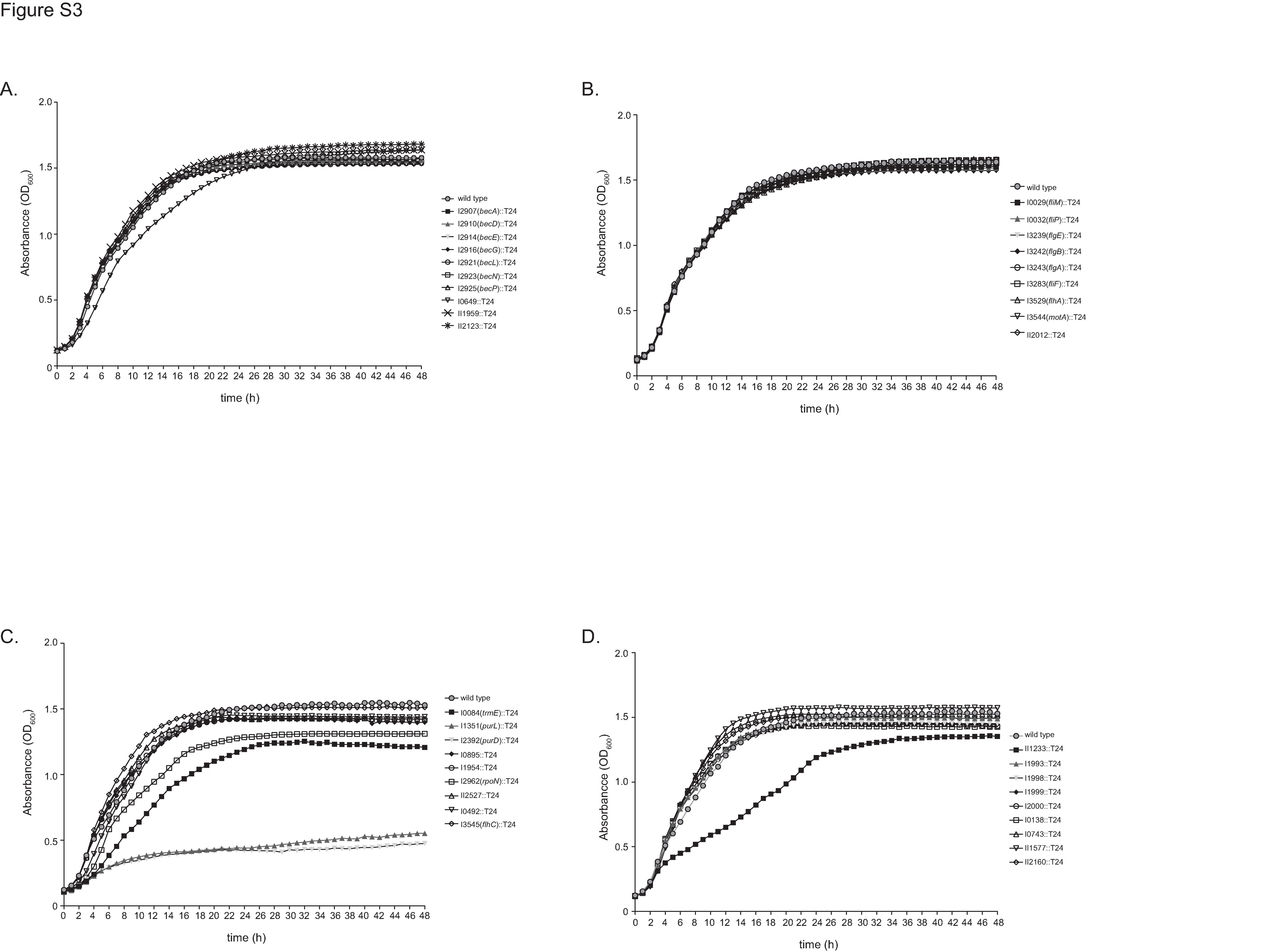

Supplement: S3 Fig — Overnight cultures were grown in LB and cultures were adjusted to a final OD600 0.1. Bacteria were grown at 37°C with shaking. Readings were taken every hour (A-D). (TIF) [file pntd.0005689.s003.tif]

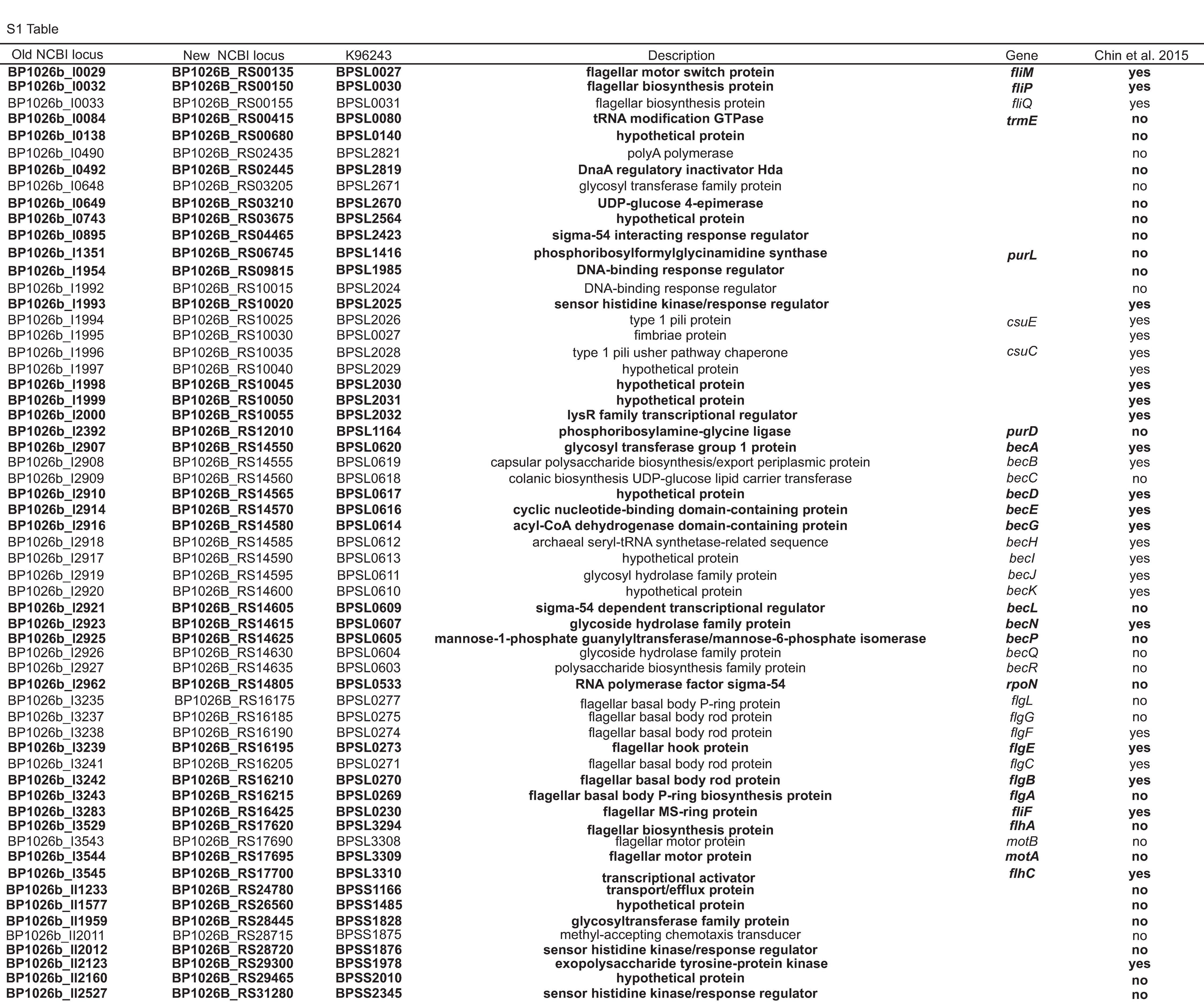

Supplement: S1 Table — Columns presented are the old NCBI gene locus, new NCBI gene locus, K96243 gene locus, gene description as found in burkholderia.com [38], gene locus, and if the gene was noted to have increased gene expression in a recent transcriptomic study [66]. Gene loci in bold represent transposon mutants that are predicted to be the first gene in an operon and were studied in detail. (TIF) [file pntd.0005689.s004.tif]

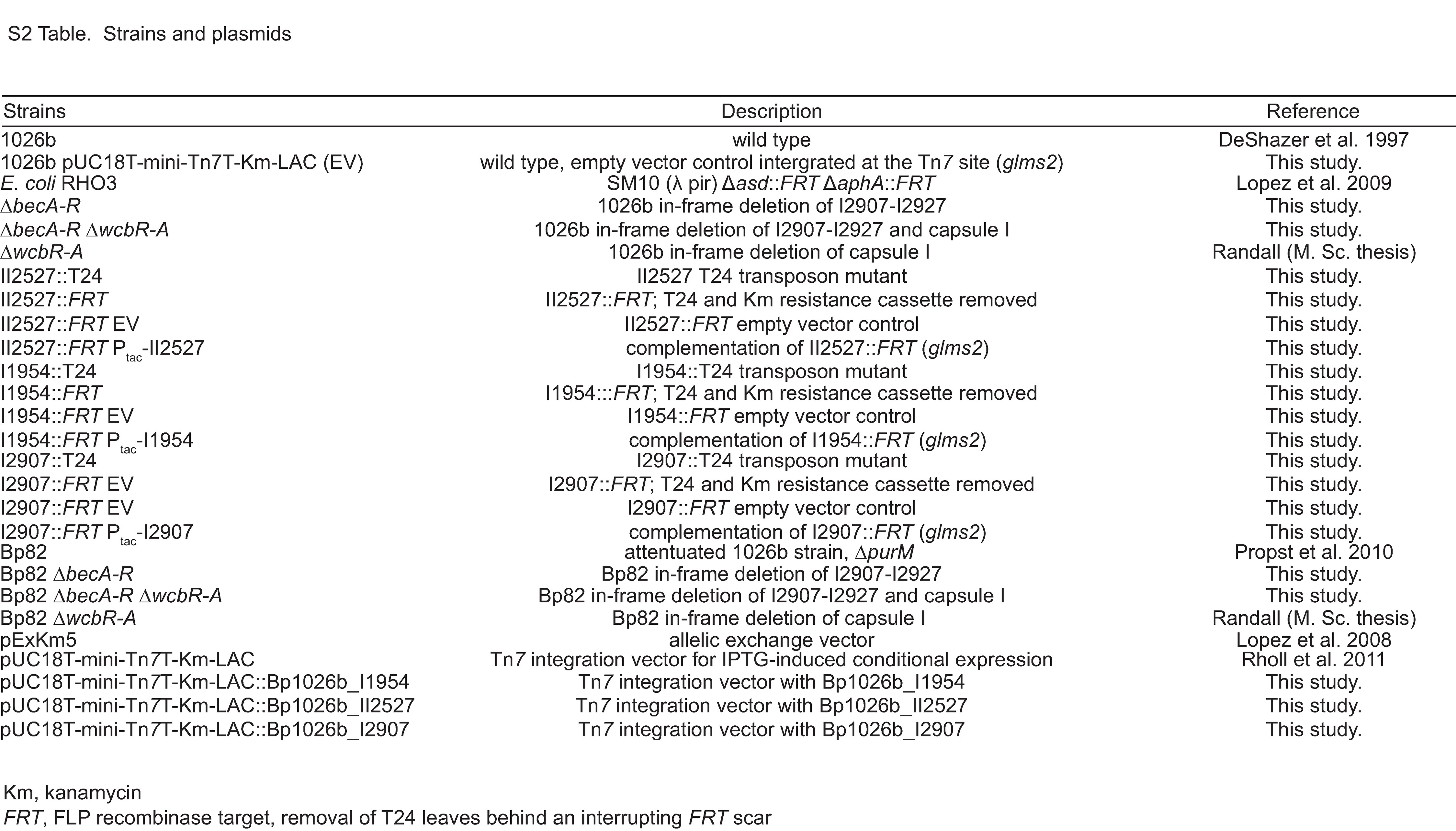

Supplement: S2 Table — (TIF) [file pntd.0005689.s005.tif]

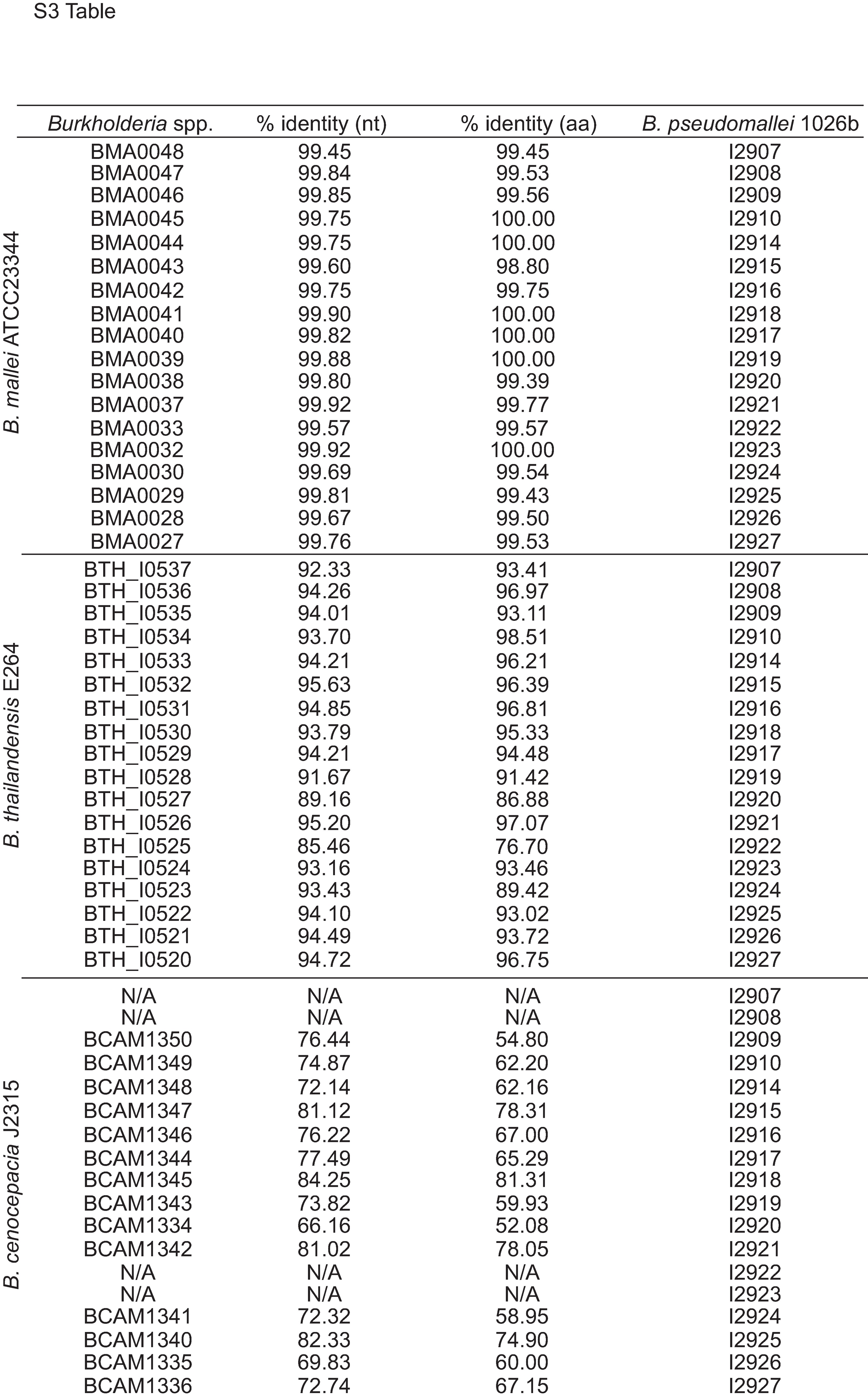

Supplement: S3 Table — (TIF) [file pntd.0005689.s006.tif]

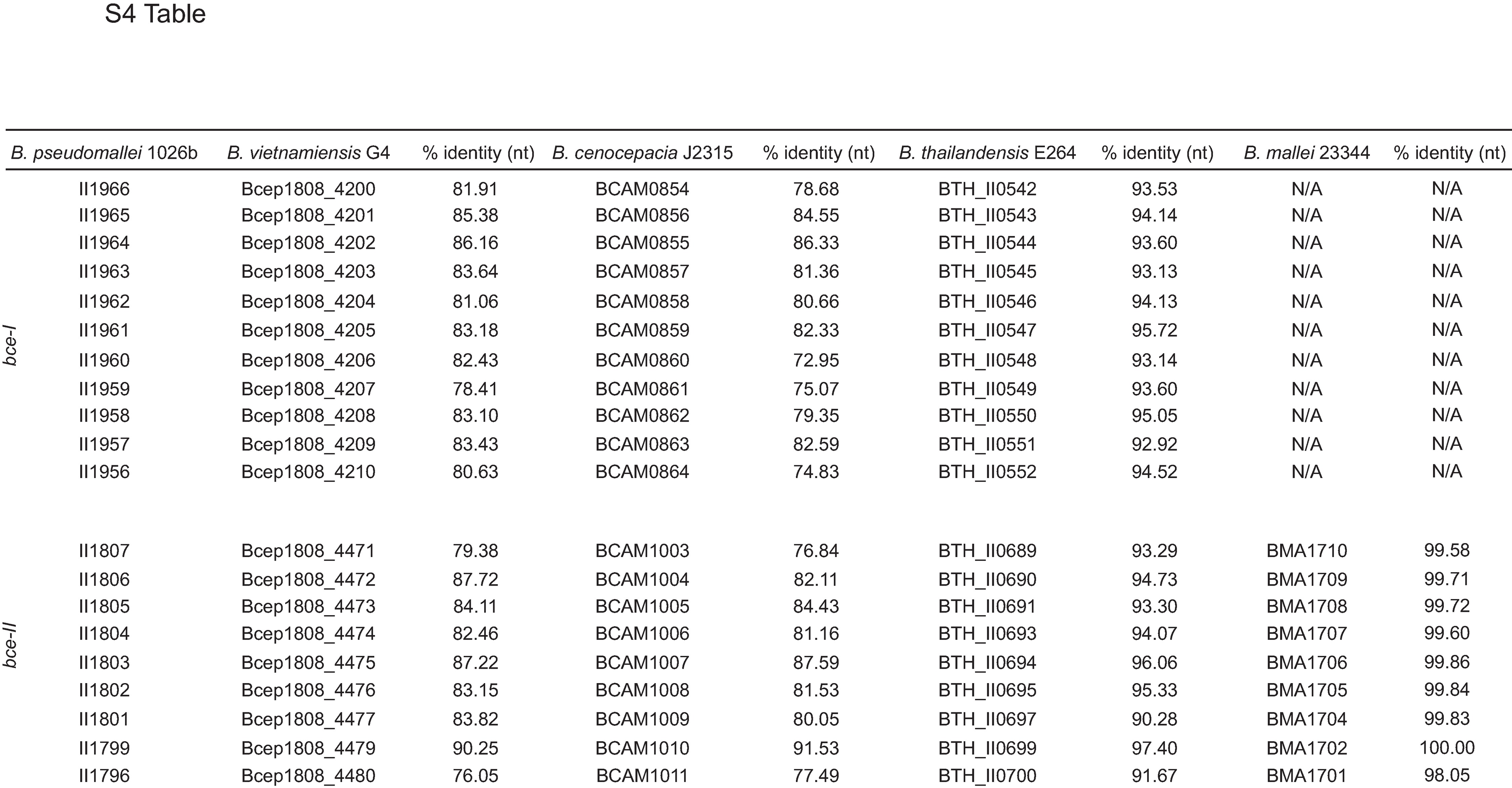

Supplement: S4 Table — (TIF) [file pntd.0005689.s007.tif]

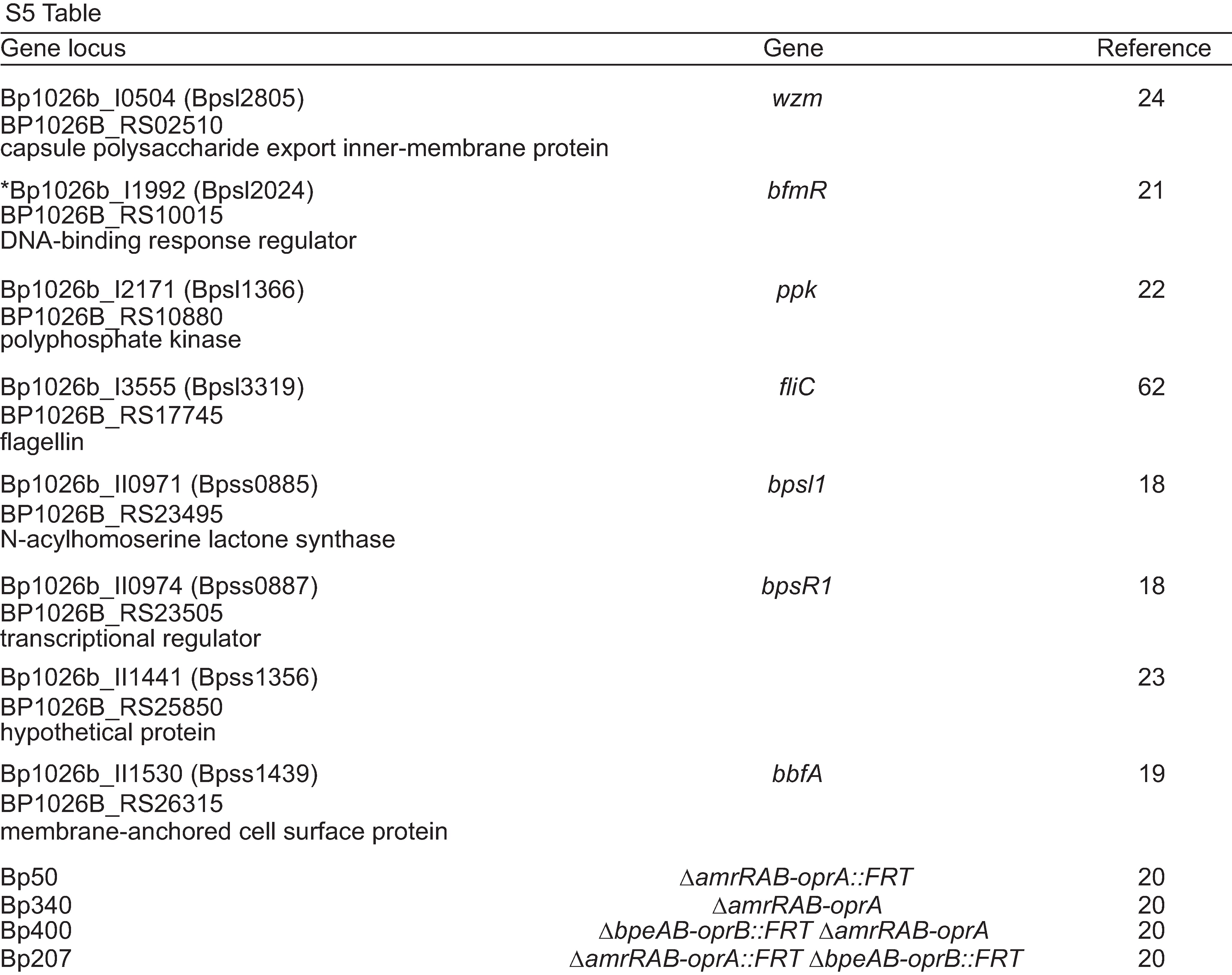

Supplement: S5 Table — The asterisk indicates that a transposon insertional mutant was identified in the screen described in the current study. (TIF) [file pntd.0005689.s008.tif]
